# Supplementary material for: Words Matter: An Antibias Workshop for Health Care Professionals to Reduce Stigmatizing Language
Source: MedEdPORTAL. 2021 Mar 2;17:11115. doi: 10.15766/mep_2374-8265.11115 (PMC7970642; doi:10.15766/mep_2374-8265.11115)
Supplement: Supplementary file 1 — Facilitator's Guide.docxPowerPoint Presentation.pptxSign-out Skit.docxMindful Language Toolkit.docxClinical Cases.docxCourse Evaluation.docx [file mep_2374-8265.11115-s001.zip › A. Facilitator's Guide.docx]

**Appendix A: Facilitator’s Guide**

Sessions objectives:

1) Explain the impact of providers’ language biases on patient care

2) Describe strategies that can be used to mitigate providers’ language biases

3) Apply strategies from the *Mindful Language Toolkit* to address stigmatizing language

The outlined workshop agenda below  is to be used as a facilitator’s guide in tandem with Appendix B, the PowerPoint Presentation. We recommend that 2-4 presenters facilitate the workshop.

| **Time**  **(mins)** | **Activity** |
| --- | --- |
| 10 | **Opening & Reflective Exercise (Appendix B): Recommend 1-2 facilitators**  Facilitators create a *Brave Space* by introducing the following ground rules:   1. Be respectful 2. Acknowledge intention and impact 3. Elevate all voices equally   For this exercise, facilitators will have audience raise their hand if they have ever seen or used the following biased language:  ●       “Sickler is here for...”; “the GSW (gunshot wound)”  ●       “Patient is faking her pain” or “medication-seeking”  ●       “Difficult” patient or family  ●       “Non-compliant” patient  ●       “He had it coming”; “he deserves this”  ●       “Patient here for ‘total body dolor’”; “pan-positive ROS”  ●       “She is just really anxious”; “she’s crazy”  Then for 3 minutes, have the audience split into pairs and reflect with a partner on past experiences where they or a colleague used stigmatizing language in their communication about a patient. The following questions can serve as a guide.  ●       What was the context in which the language was used?  ●       Did you recognize your bias at that time?  ●       Did it affect how you cared for the patient?  ●       What could you have done differently?  Ask for volunteers to share their reflection with the group– *can be optional if the audience feels uncomfortable or facilitators can take this moment and share their own experiences when they have used biased language.* |
| 5 | **Skit of Clinical Sign-out: Two facilitators will demonstrate the use of stigmatizing language in a role play for the audience (Appendix C)**  Examples of biased language from the sign-out:  ●       **“**Just defer to the daytime; “she’s really anxious - don’t worry about it”; “you’ll probably get called in tonight”; “worried well”  ●       “Crazy”; “difficult/a nightmare”; “I would avoid that room tonight if you can”; “I heard Mom’s boyfriend just got out of jail”; rolling eyes  ●       Poorly controlled DM patient - coming in having overdosed on insulin, with glucose 30; dismissed  As a large group, facilitators will discuss the following questions with audience:   - Did you recognize bias in the language used in their role play? - How did that sign out make you feel? - In what contexts have you encountered similar stigmatizing language? - Did it affect patient care? If so, how? - What barriers are there to mitigating this? |
| 10 | **Brief Didactic** **(Appendix B): Recommend 1-2 facilitators**  Define language biases.  Describe how language bias can affect patient care and health outcomes. |
| 15 | **Small Group Activity and Discussion (Appendix E): Recommend 1-2 facilitators**  Audience will divide into small groups. Each group will assigned one of two clinical cases and discuss the following questions for 10 minutes:  ●       What language biases were present in each case?  ●       How did you identify them?  ●       How do you think it could affect patient care?  ●       What challenges do providers face when trying to avoid stigmatizing language? *Of note, these cases may be adapted to any clinical discipline. Do not distribute the clinical cases with the revised language at this time.*  If choosing to use an online platform such as Sli.do.™, have people text the biased language identified in the case to display on word cloud. Alternatively, participants can write biased language identified onto parchment paper or flip chart.  Facilitators will then debrief the activity with the large group for 5 minutes. |
| 10 | **Brief Didactic (Appendix B & D): Recommend 1-2 facilitators**  Facilitators present a framework for identifying biased language and how to mitigate and replace them with unbiased descriptions. Facilitators can distribute printed copies of Appendix D, *The Mindful Language Toolkit, which will be used in the next activity.* |
| 15 | **Small Group Practice Exercise (Appendix E): Recommend 1-2 facilitators**  Audience will return to their previous small group. Using the toolkit provided (Appendix D), each group will rewrite their case with improved language, reflecting on the following questions.  ●       How did you change the language to reduce bias?  ●       How did you facilitate others’ recognition of the bias?  ●       What feedback strategy did you use? |
| 20 | **Large Group Discussion (Appendix B & E): Recommend 1-2 facilitators**  Facilitators will now ask participants to share their revised cases and encourage reflection.  ●       What barriers did people encounter with this exercise?  *Facilitators can then distribute the revised clinical cases (Appendix E). We recommend facilitators be explicit that these revised cases are not perfect and encourage learners to reflect what aspects can still be improved.*  *Lastly, facilitators will facilitate discussion on the following questions with the audience:*  ●       Do you find this issue present at your hospital or institution?  ●      What challenges would you face to implementing change?  Any strategies to combat these barriers to create change?  ●       How will you approach colleagues who use biased language? How about learners? Supervisors? (Reference Graves, et al.’s *MedEdPortal* workshop)[^1^](https://www.zotero.org/google-docs/?rkt05X) |
| 5 | **Conclusions**  Summarize takeaway points and distribute workshop evaluations. |
