# Supplementary material for: Words Matter: An Antibias Workshop for Health Care Professionals to Reduce Stigmatizing Language
Source: MedEdPORTAL. 2021 Mar 2;17:11115. doi: 10.15766/mep_2374-8265.11115 (PMC7970642; doi:10.15766/mep_2374-8265.11115)
Supplement: Supplementary file 1 — Facilitator's Guide.docxPowerPoint Presentation.pptxSign-out Skit.docxMindful Language Toolkit.docxClinical Cases.docxCourse Evaluation.docx [file mep_2374-8265.11115-s001.zip › B. PowerPoint Presentation.pptx]

## Slide 1
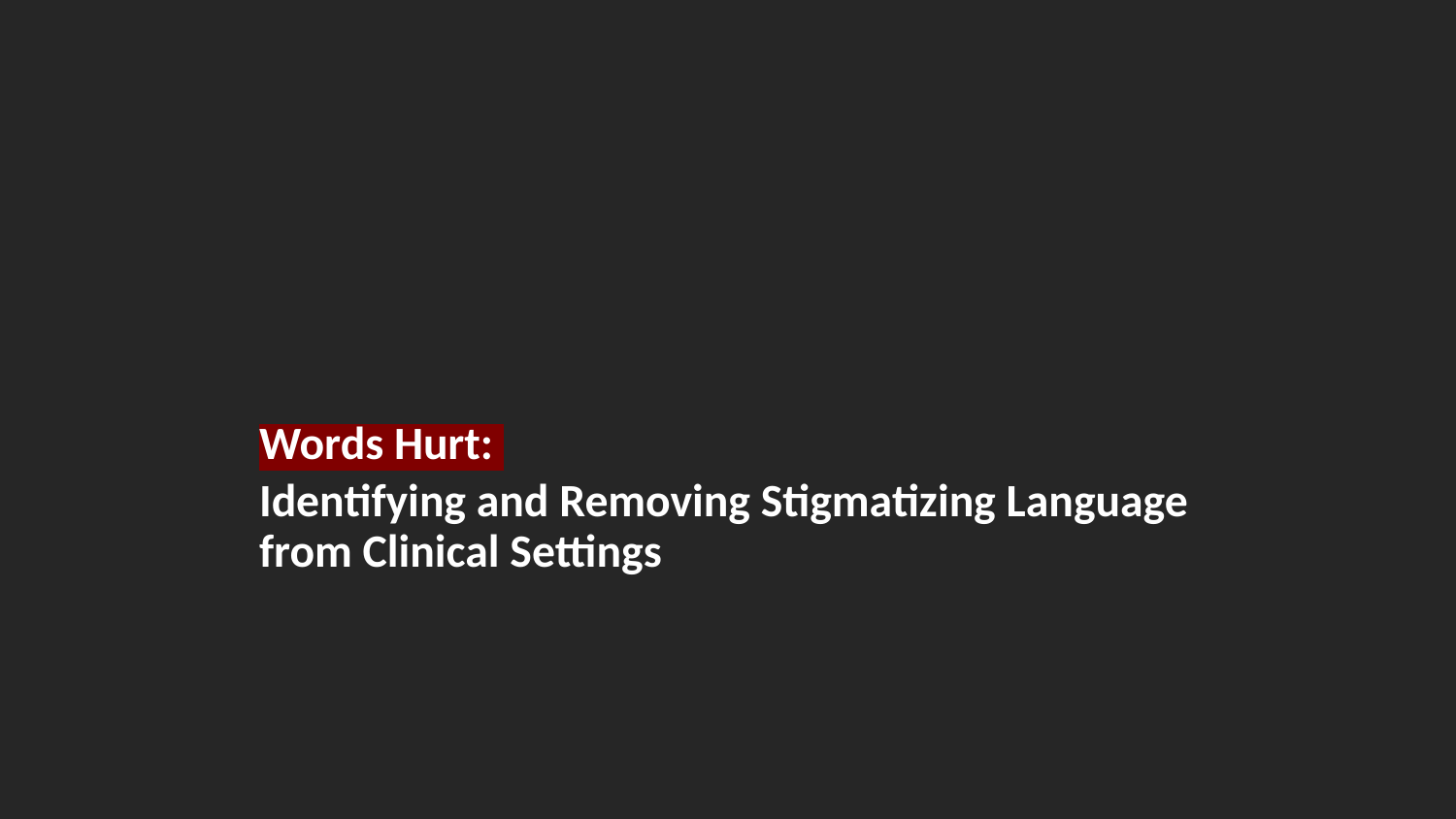

# Words Hurt: Identifying and Removing Stigmatizing Language from Clinical Settings

## Slide 2
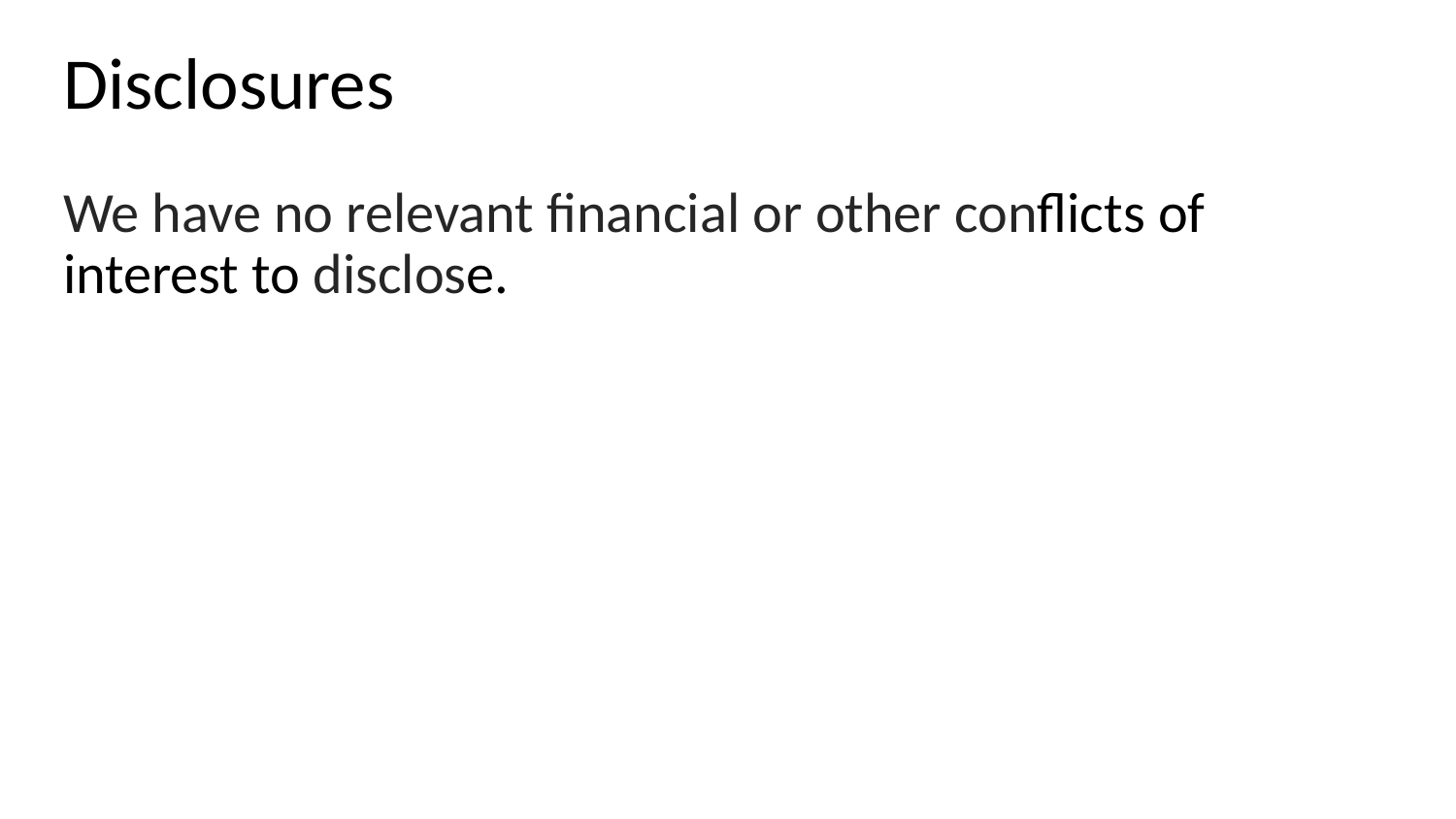

# Disclosures
We have no relevant financial or other conflicts of interest to disclose.

## Slide 3
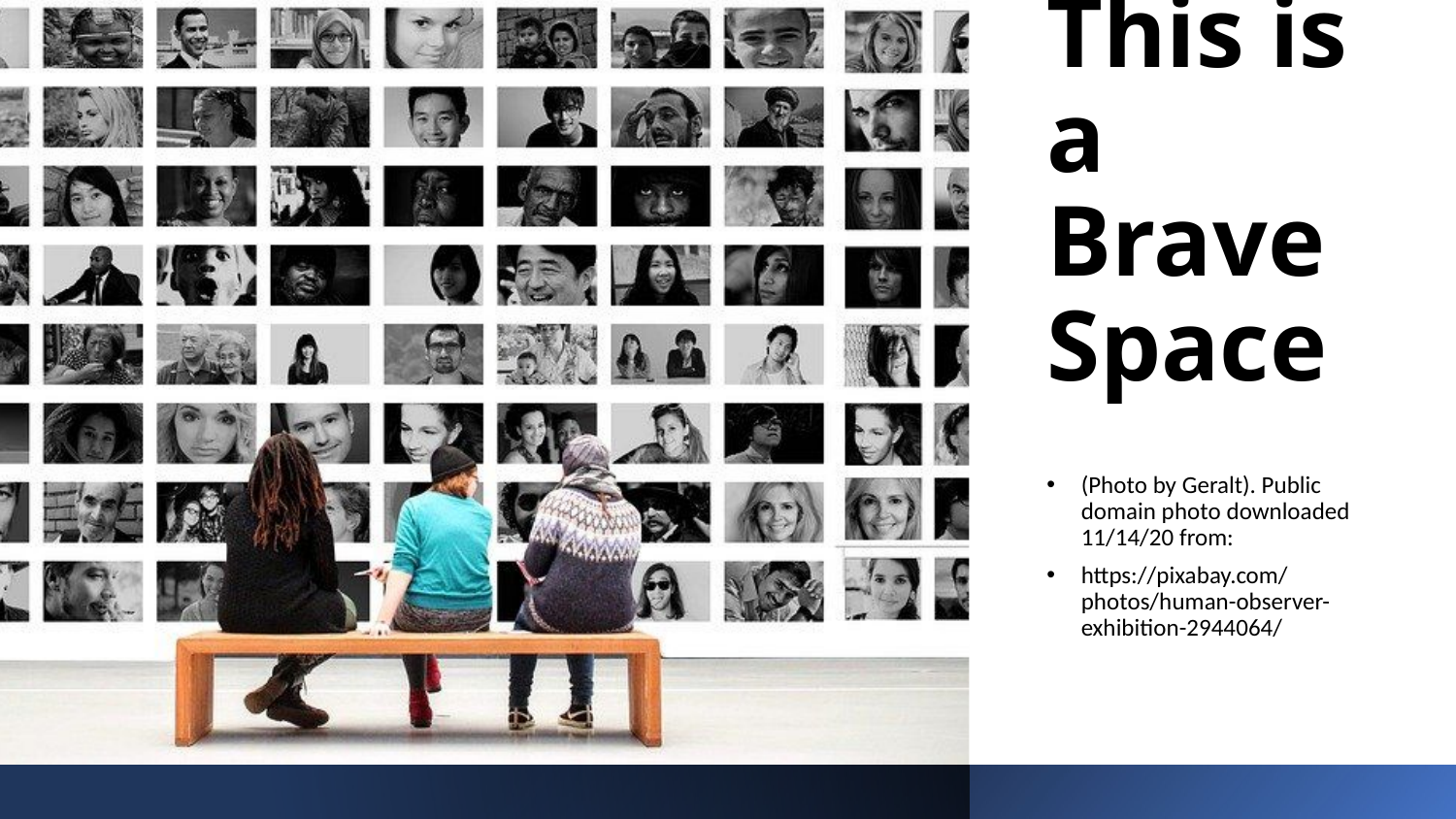

This is a Brave Space
(Photo by Geralt). Public domain photo downloaded 11/14/20 from:
https://pixabay.com/photos/human-observer-exhibition-2944064/

## Slide 4
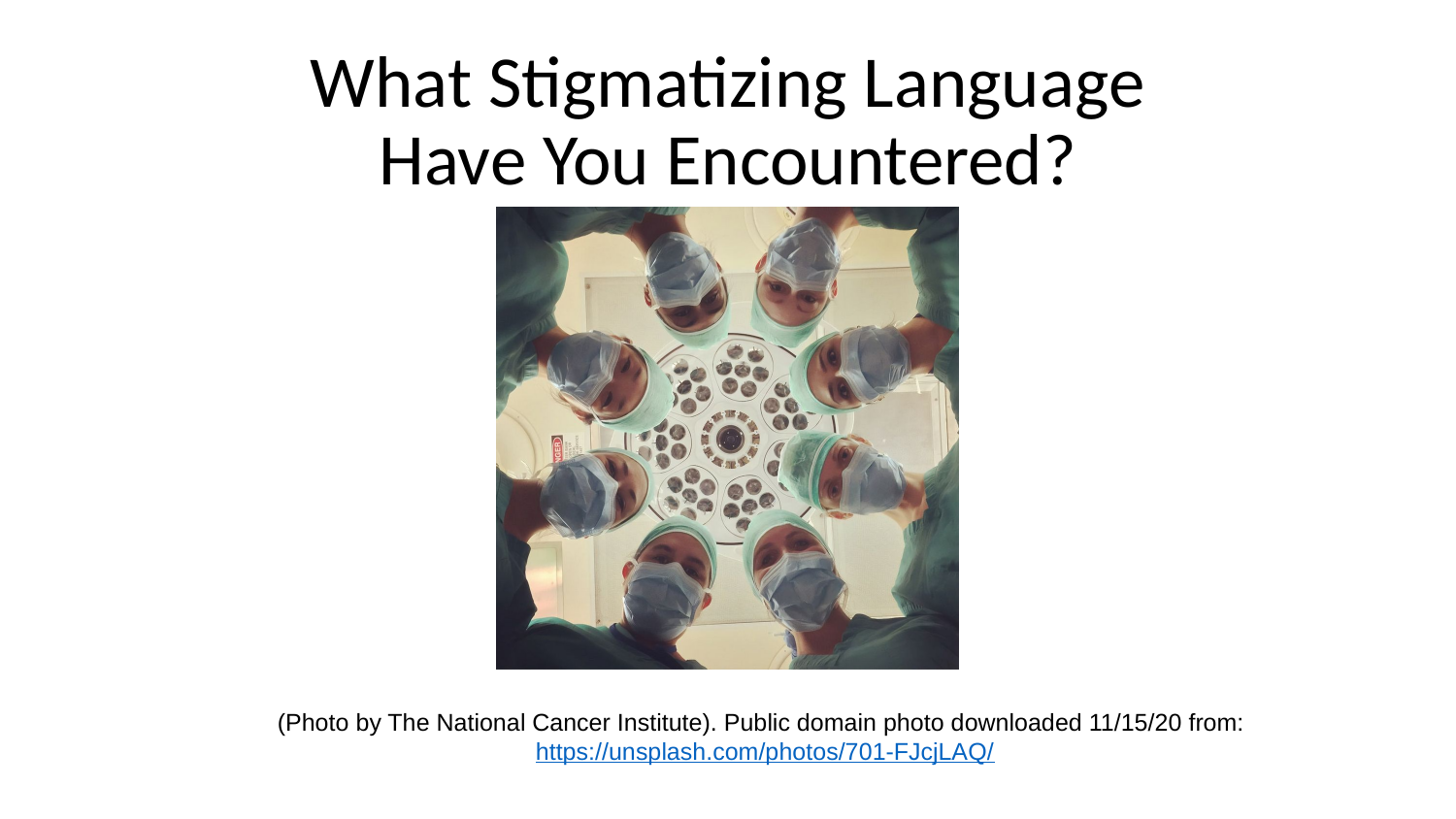

# What Stigmatizing Language Have You Encountered?
(Photo by The National Cancer Institute). Public domain photo downloaded 11/15/20 from:
https://unsplash.com/photos/701-FJcjLAQ/

## Slide 5
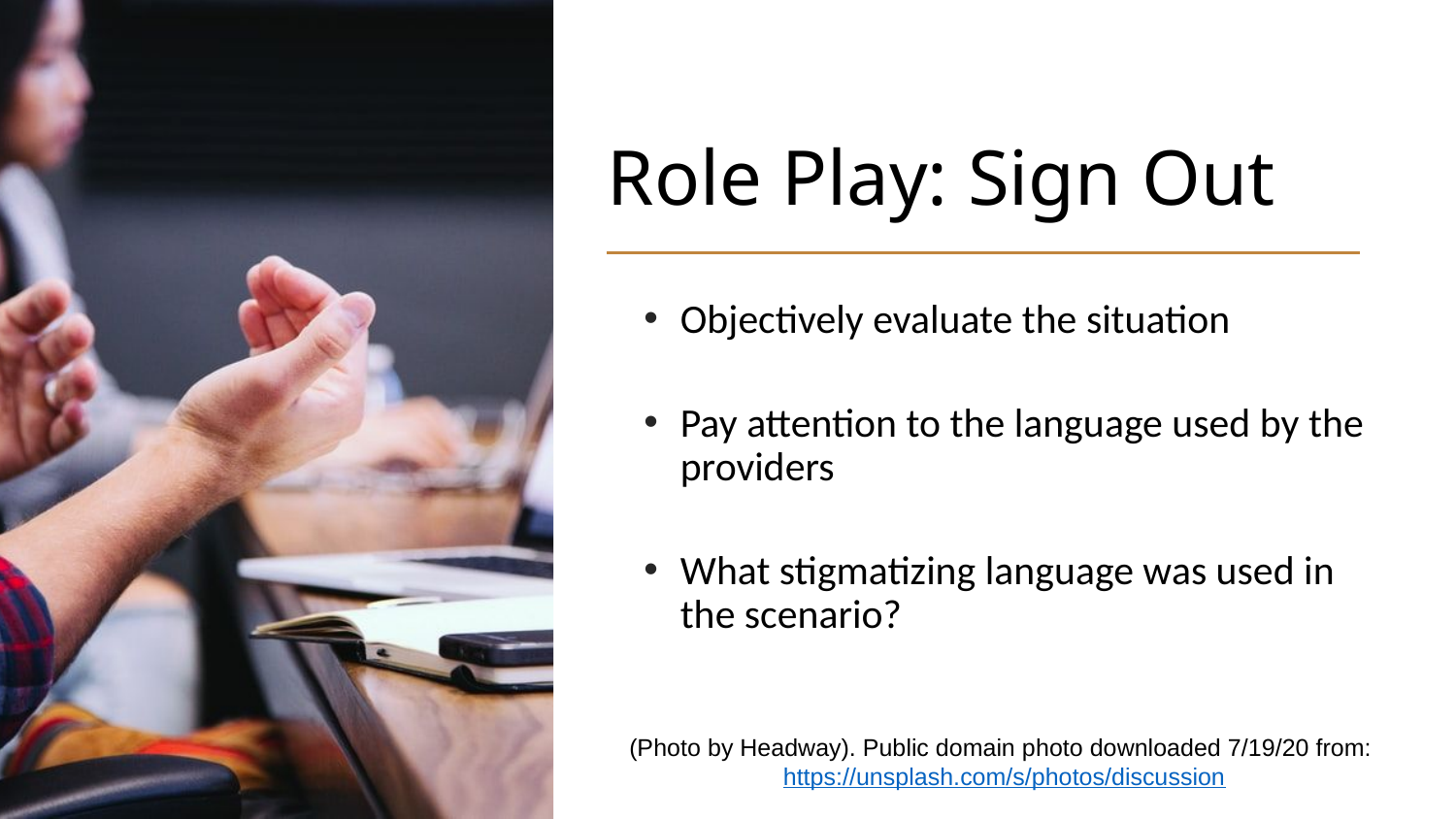

# Role Play: Sign Out
Objectively evaluate the situation
Pay attention to the language used by the providers
What stigmatizing language was used in the scenario?
(Photo by Headway). Public domain photo downloaded 7/19/20 from:
https://unsplash.com/s/photos/discussion

## Slide 6
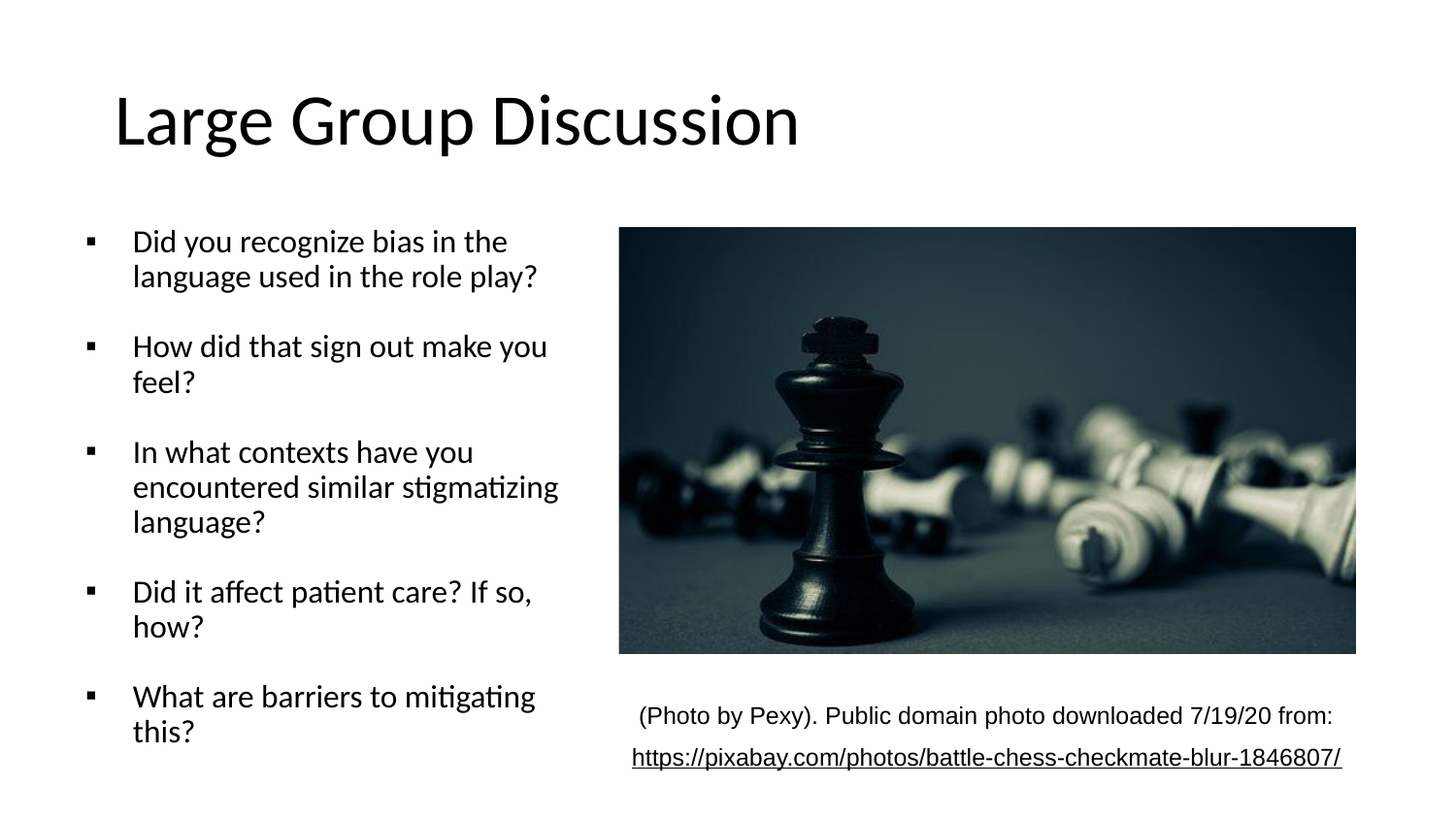

# Large Group Discussion
Did you recognize bias in the language used in the role play?
How did that sign out make you feel?
In what contexts have you encountered similar stigmatizing language?
Did it affect patient care? If so, how?
What are barriers to mitigating this?
(Photo by Pexy). Public domain photo downloaded 7/19/20 from:
https://pixabay.com/photos/battle-chess-checkmate-blur-1846807/

## Slide 7
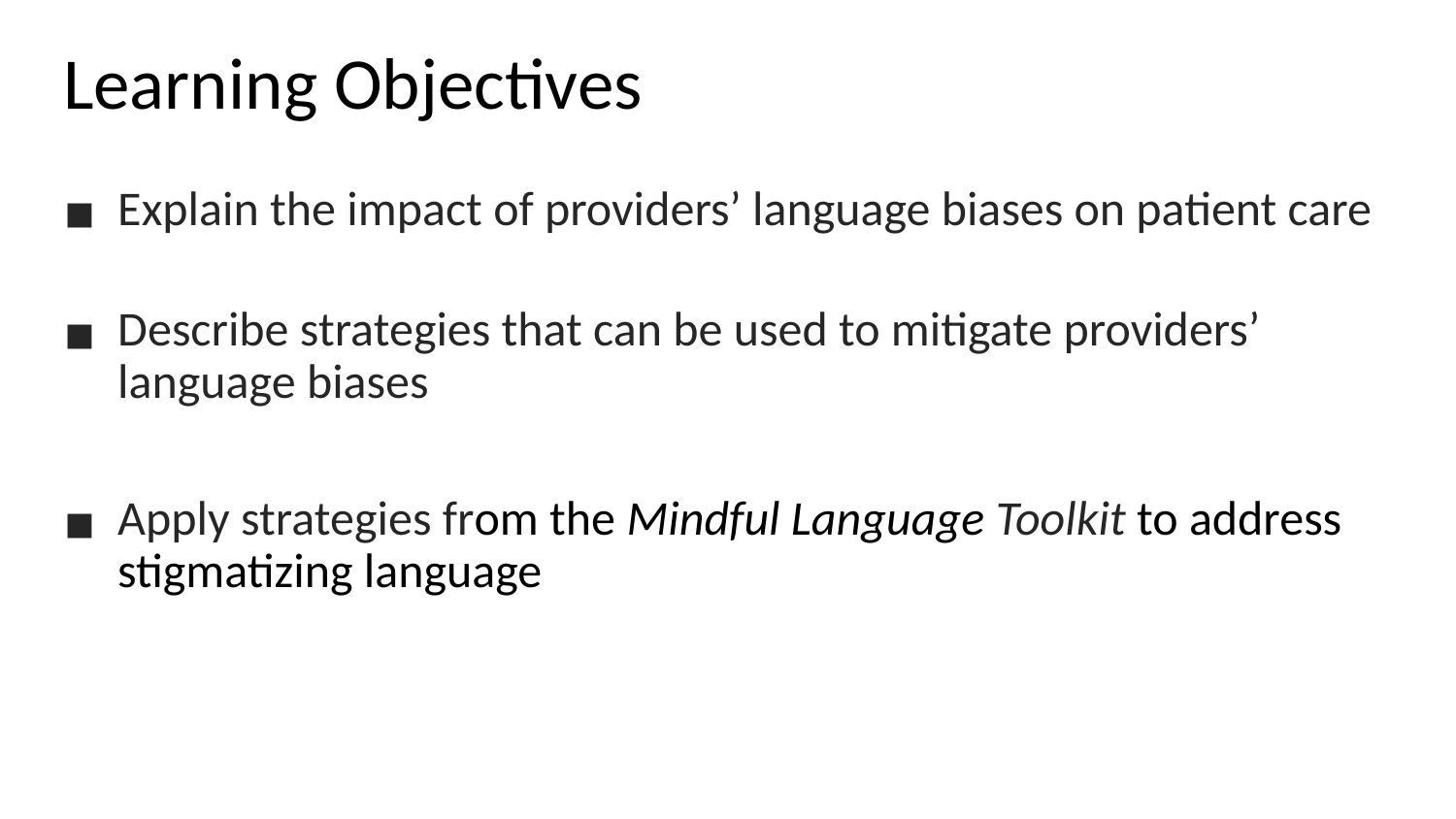

# Learning Objectives
Explain the impact of providers’ language biases on patient care
Describe strategies that can be used to mitigate providers’ language biases
Apply strategies from the Mindful Language Toolkit to address stigmatizing language

## Slide 8
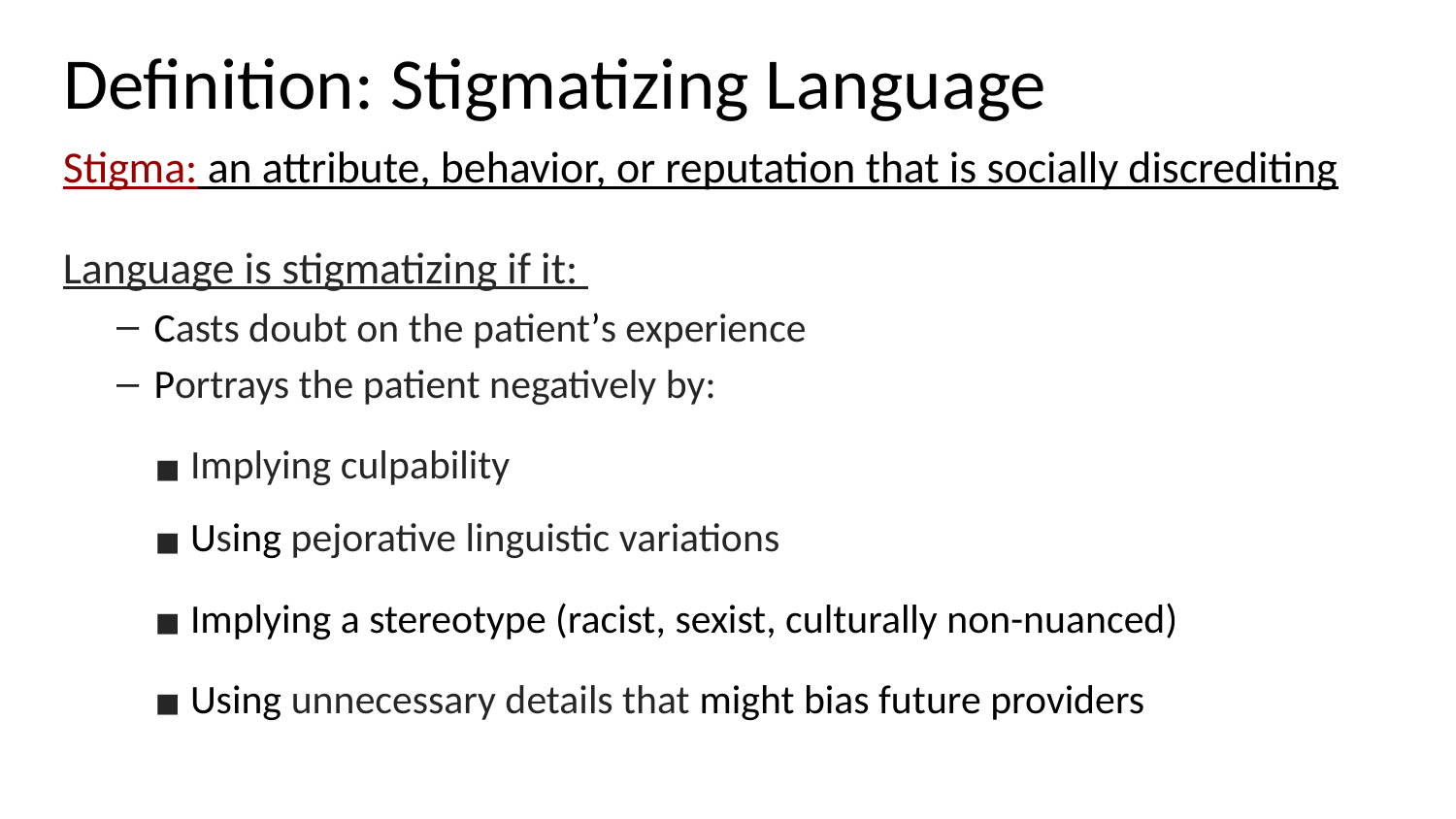

# Definition: Stigmatizing Language
Stigma: an attribute, behavior, or reputation that is socially discrediting
Language is stigmatizing if it:
Casts doubt on the patient’s experience
Portrays the patient negatively by:
Implying culpability
Using pejorative linguistic variations
Implying a stereotype (racist, sexist, culturally non-nuanced)
Using unnecessary details that might bias future providers

## Slide 9
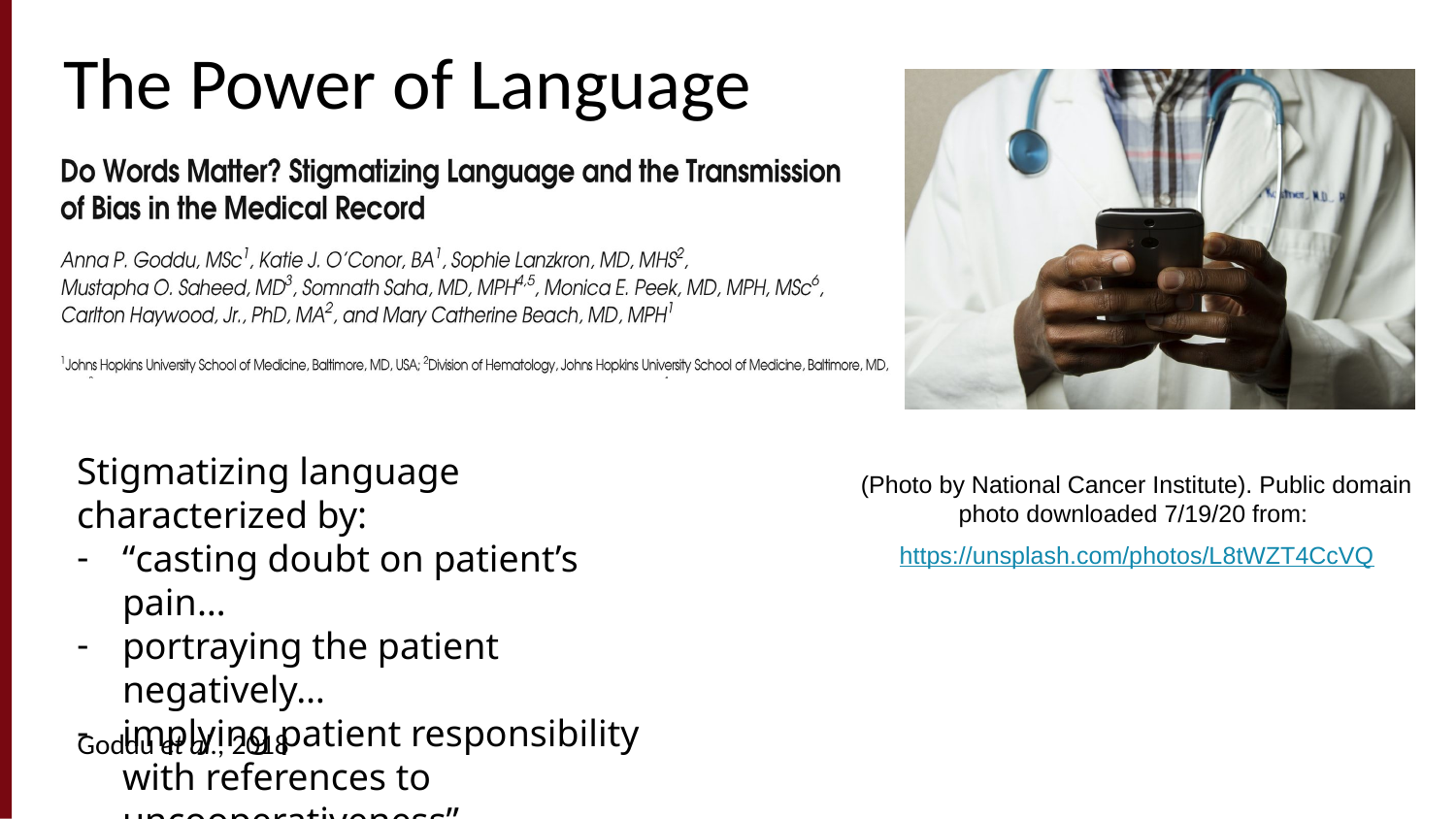

# The Power of Language
Stigmatizing language characterized by:
“casting doubt on patient’s pain…
portraying the patient negatively…
implying patient responsibility with references to uncooperativeness”
(Photo by National Cancer Institute). Public domain photo downloaded 7/19/20 from:
https://unsplash.com/photos/L8tWZT4CcVQ
Goddu et al., 2018

## Slide 10
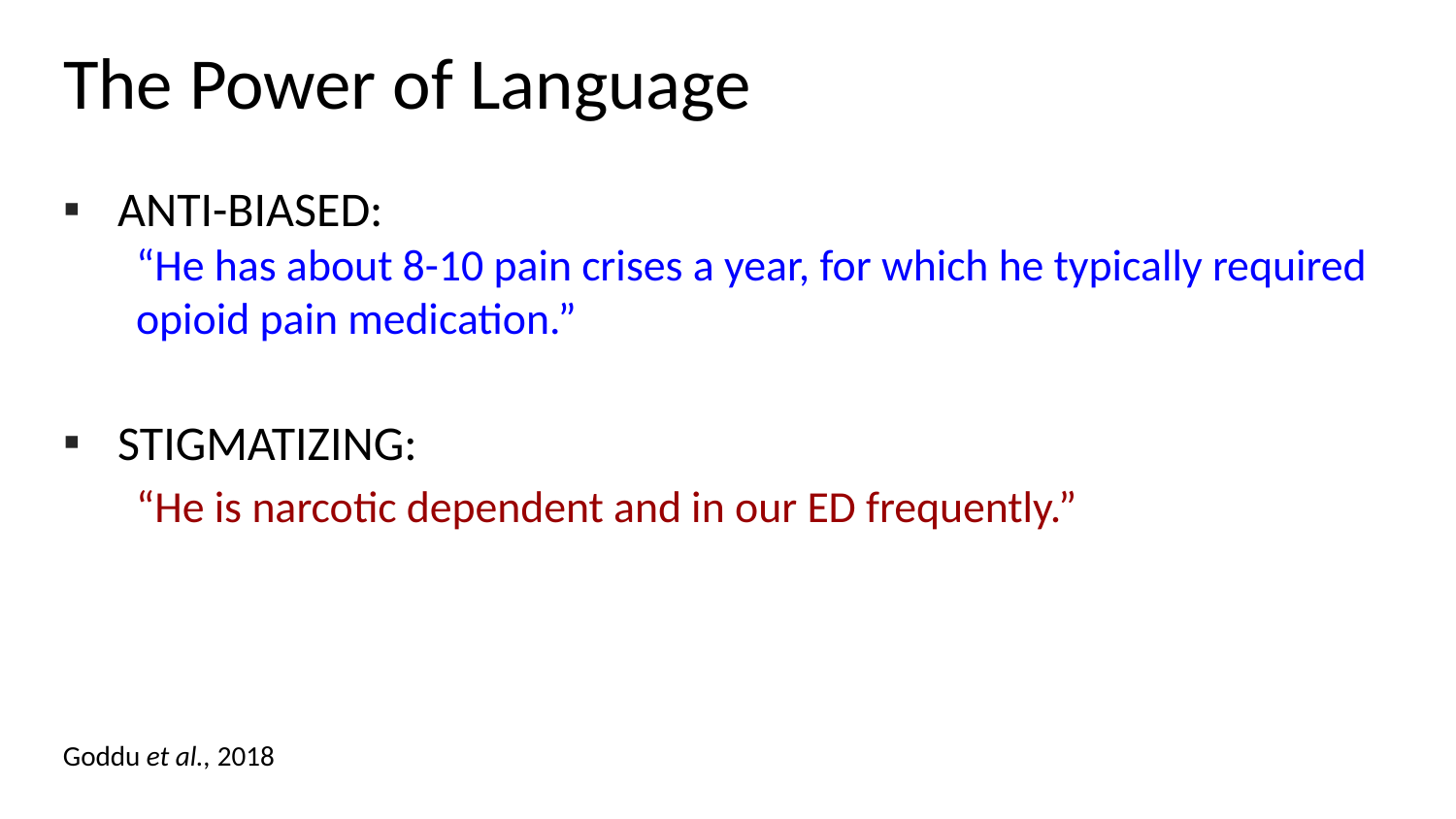

# The Power of Language
ANTI-BIASED:
“He has about 8-10 pain crises a year, for which he typically required opioid pain medication.”
STIGMATIZING:
“He is narcotic dependent and in our ED frequently.”
Goddu et al., 2018

## Slide 11
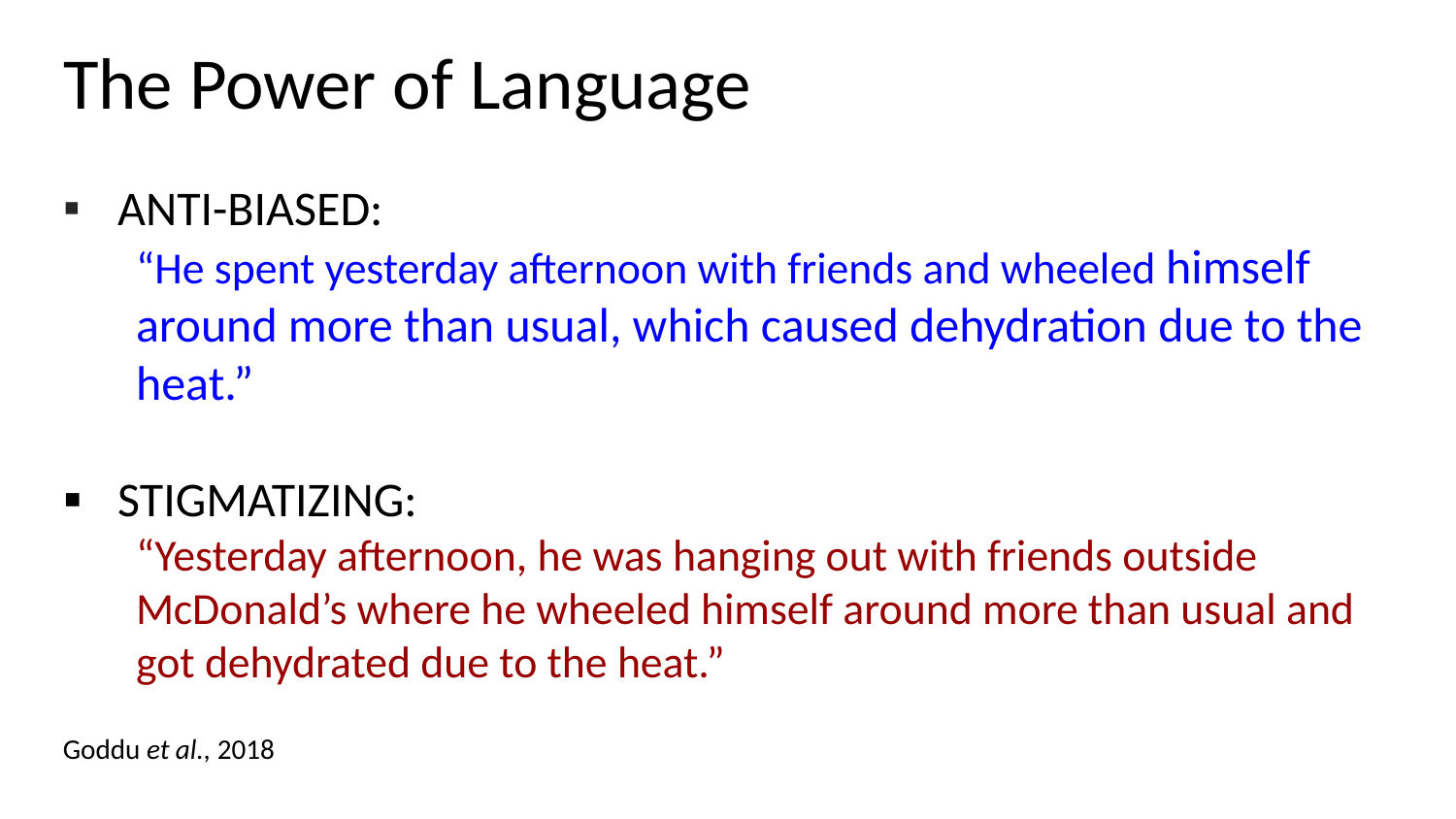

# The Power of Language
ANTI-BIASED:
“He spent yesterday afternoon with friends and wheeled himself around more than usual, which caused dehydration due to the heat.”
STIGMATIZING:
“Yesterday afternoon, he was hanging out with friends outside McDonald’s where he wheeled himself around more than usual and got dehydrated due to the heat.”
Goddu et al., 2018

## Slide 12
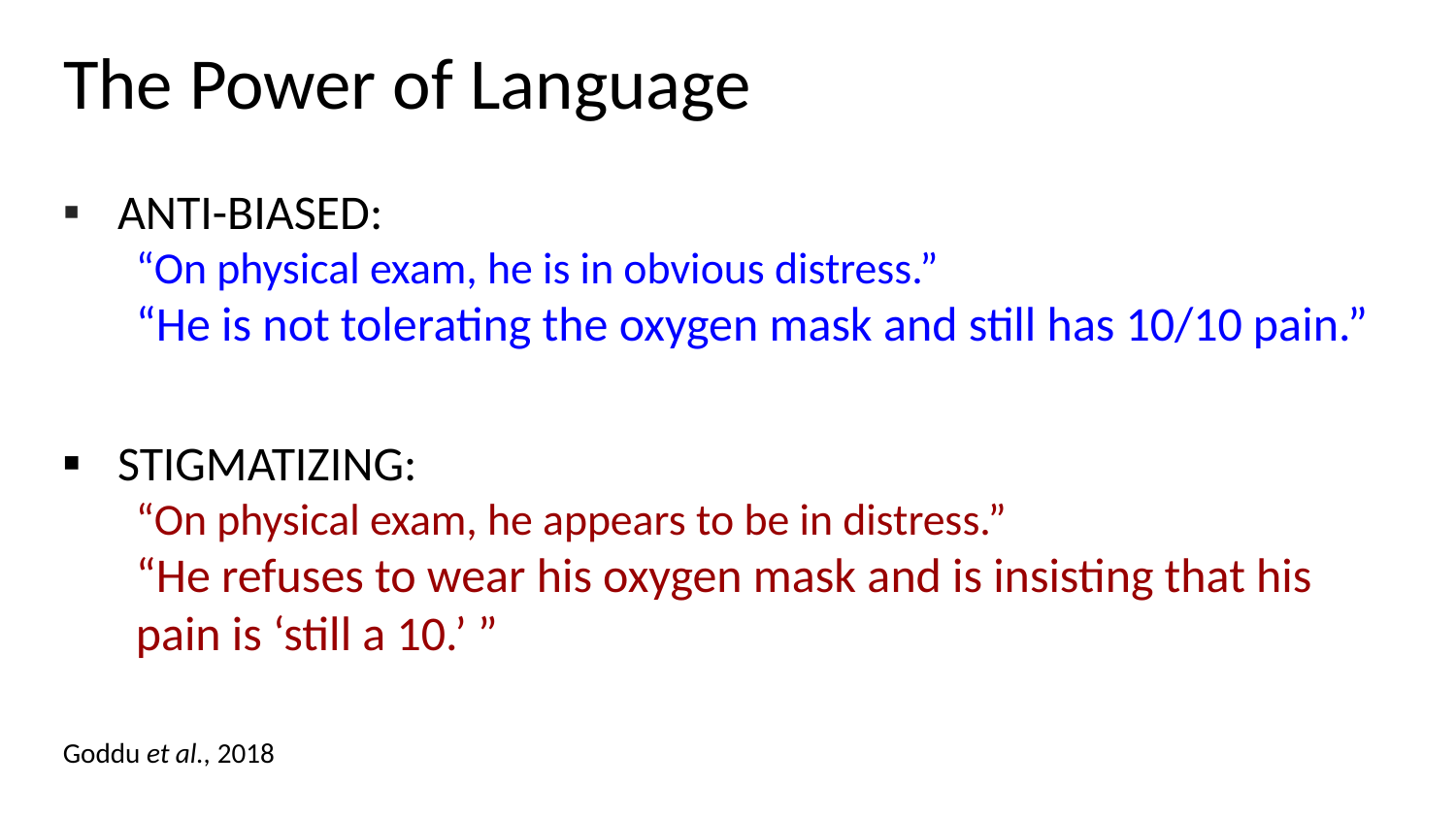

# The Power of Language
ANTI-BIASED:
“On physical exam, he is in obvious distress.”
“He is not tolerating the oxygen mask and still has 10/10 pain.”
STIGMATIZING:
“On physical exam, he appears to be in distress.”
“He refuses to wear his oxygen mask and is insisting that his pain is ‘still a 10.’ ”
Goddu et al., 2018

## Slide 13
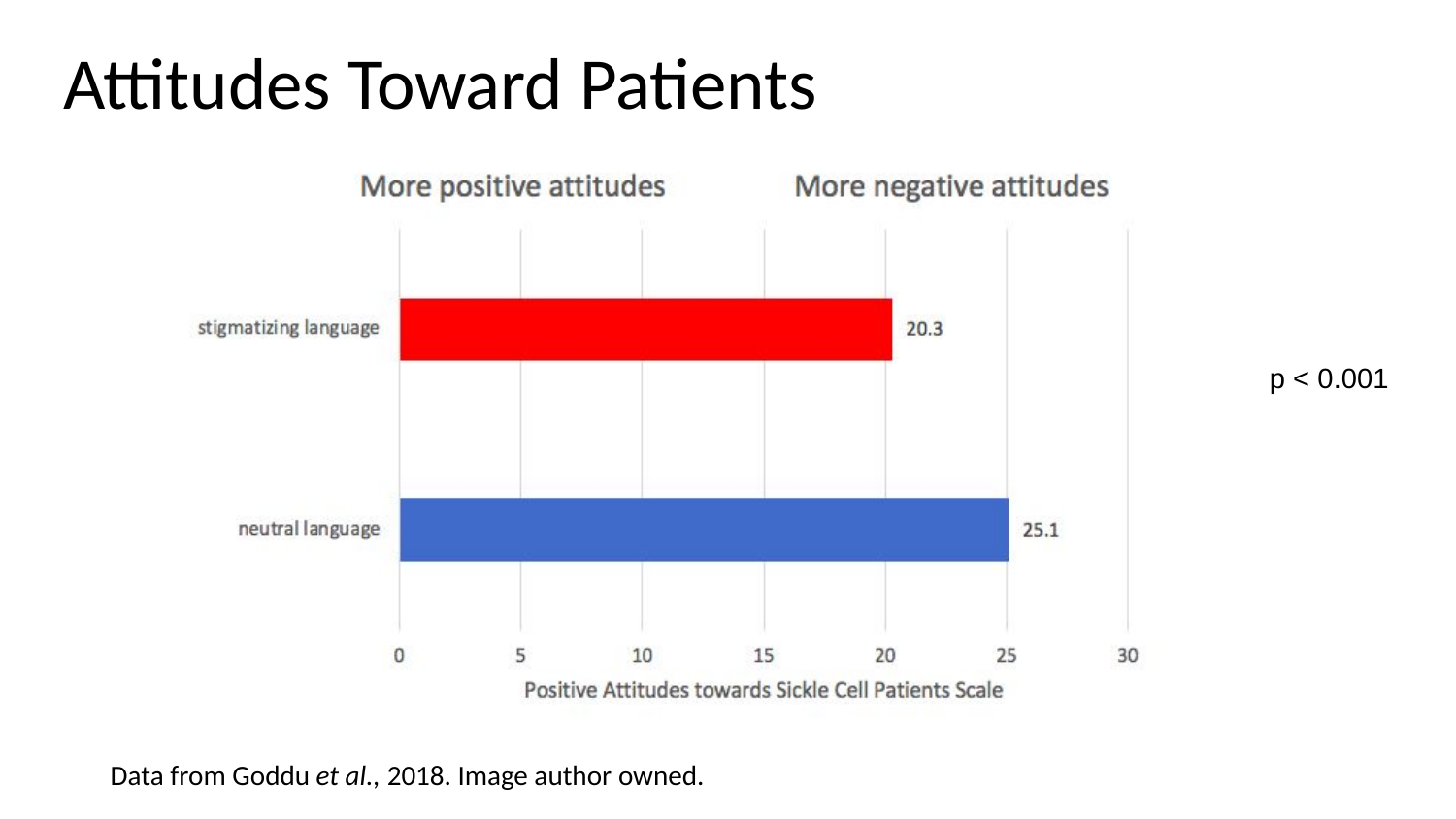

# Attitudes Toward Patients
p < 0.001
Data from Goddu et al., 2018. Image author owned.

## Slide 14
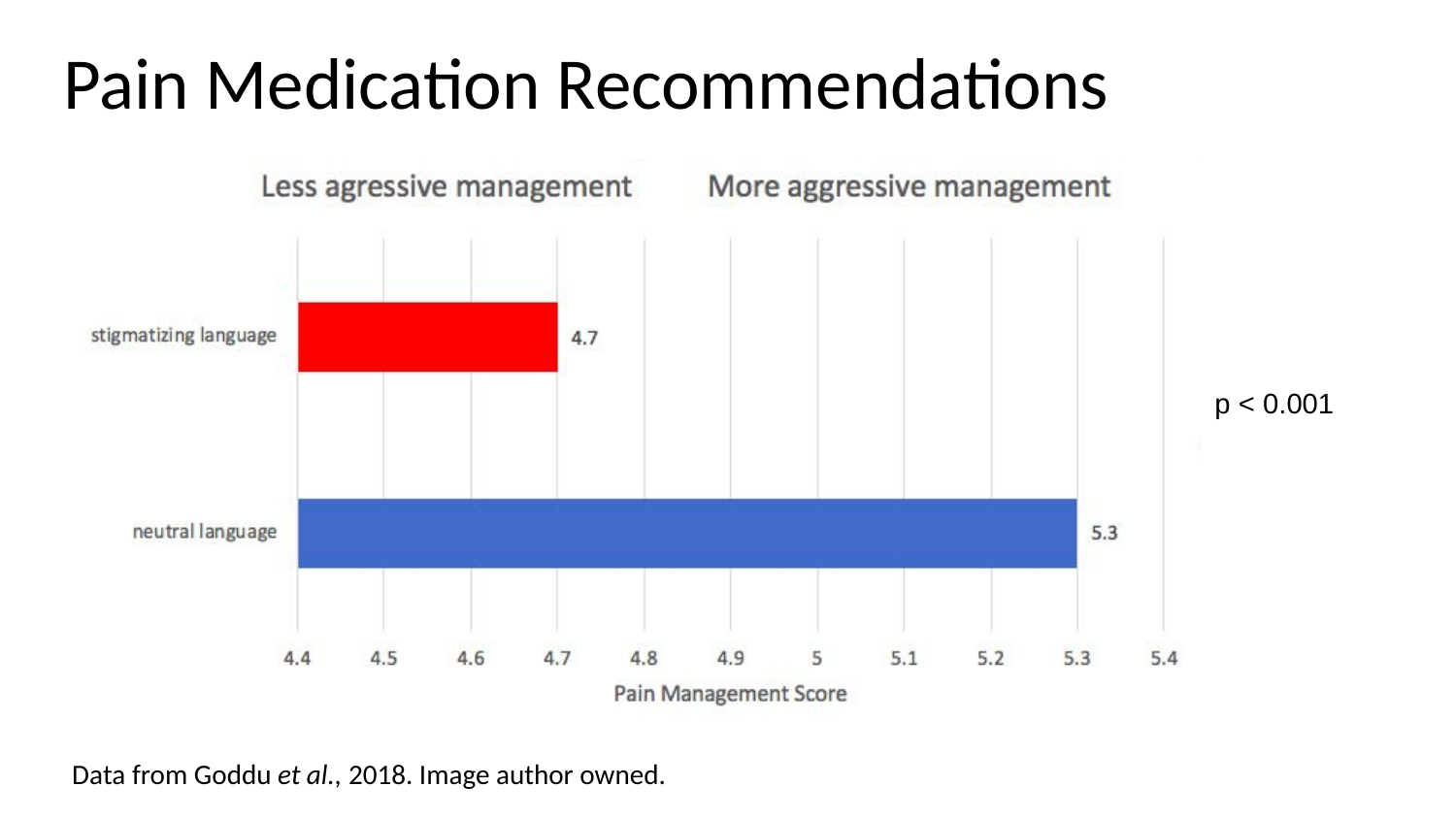

# Pain Medication Recommendations
p < 0.001
Data from Goddu et al., 2018. Image author owned.

## Slide 15
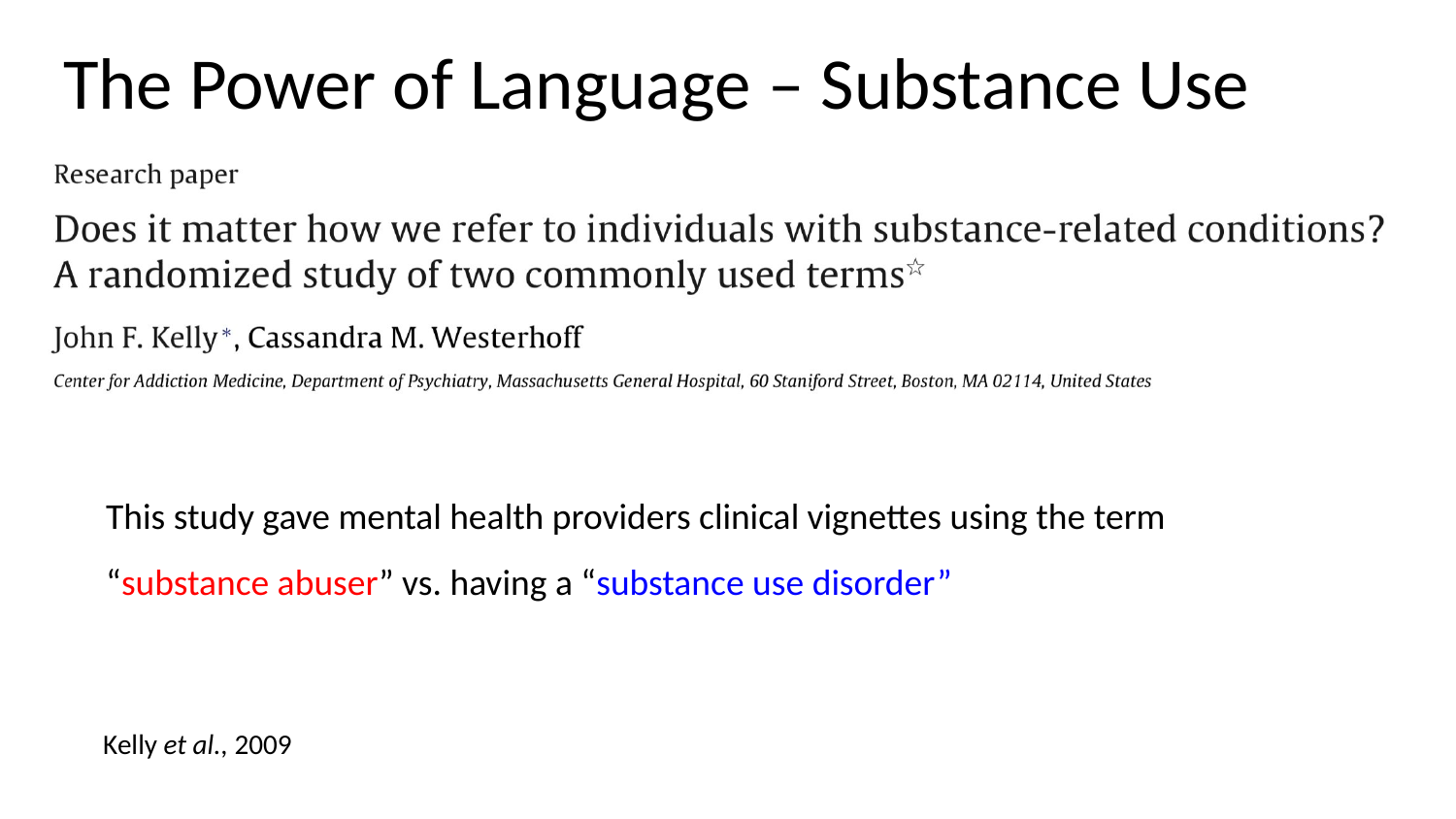

# The Power of Language – Substance Use
This study gave mental health providers clinical vignettes using the term “substance abuser” vs. having a “substance use disorder”
Kelly et al., 2009

## Slide 16
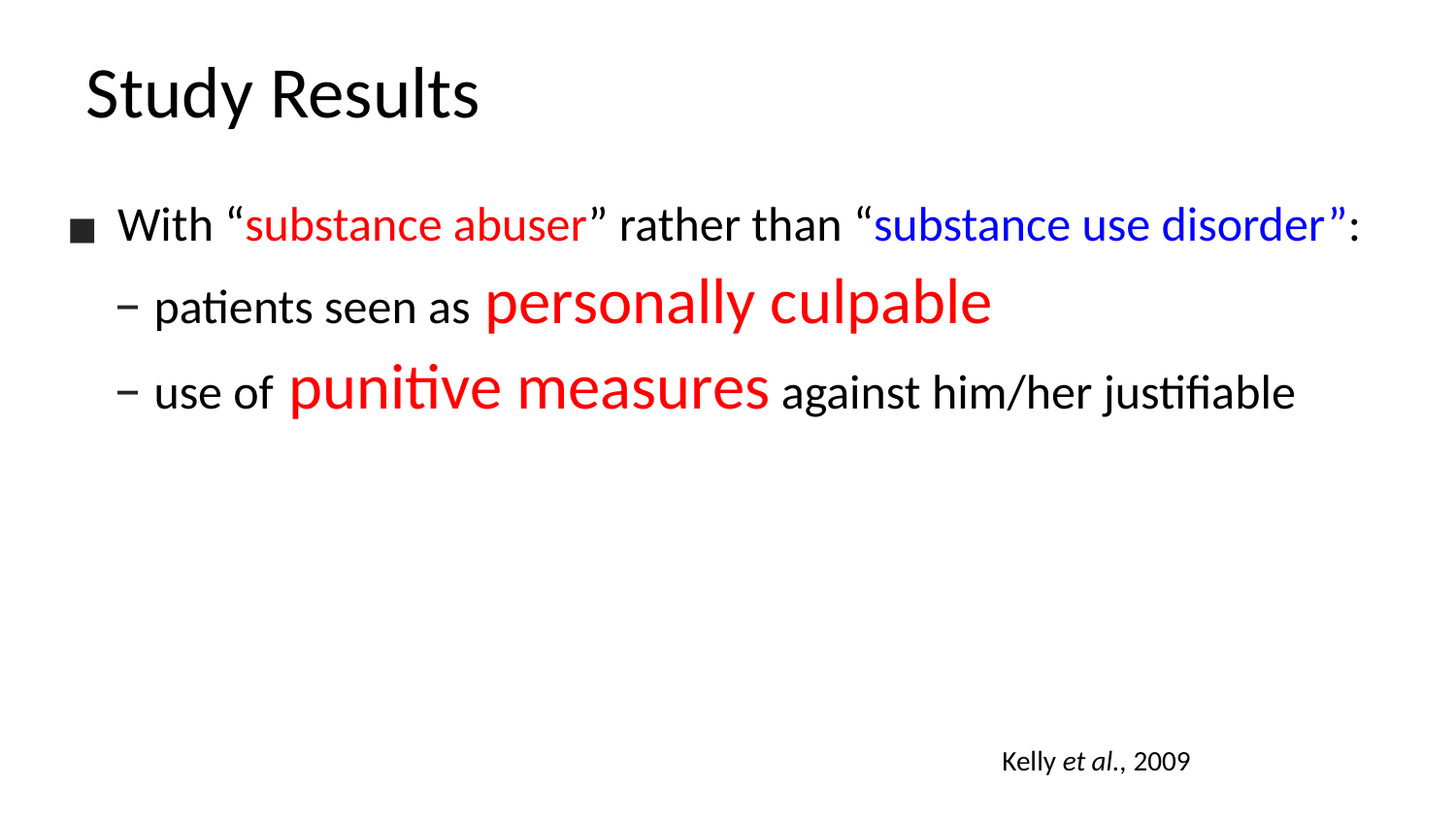

Study Results
With “substance abuser” rather than “substance use disorder”:
patients seen as personally culpable
use of punitive measures against him/her justifiable
Kelly et al., 2009

## Slide 17
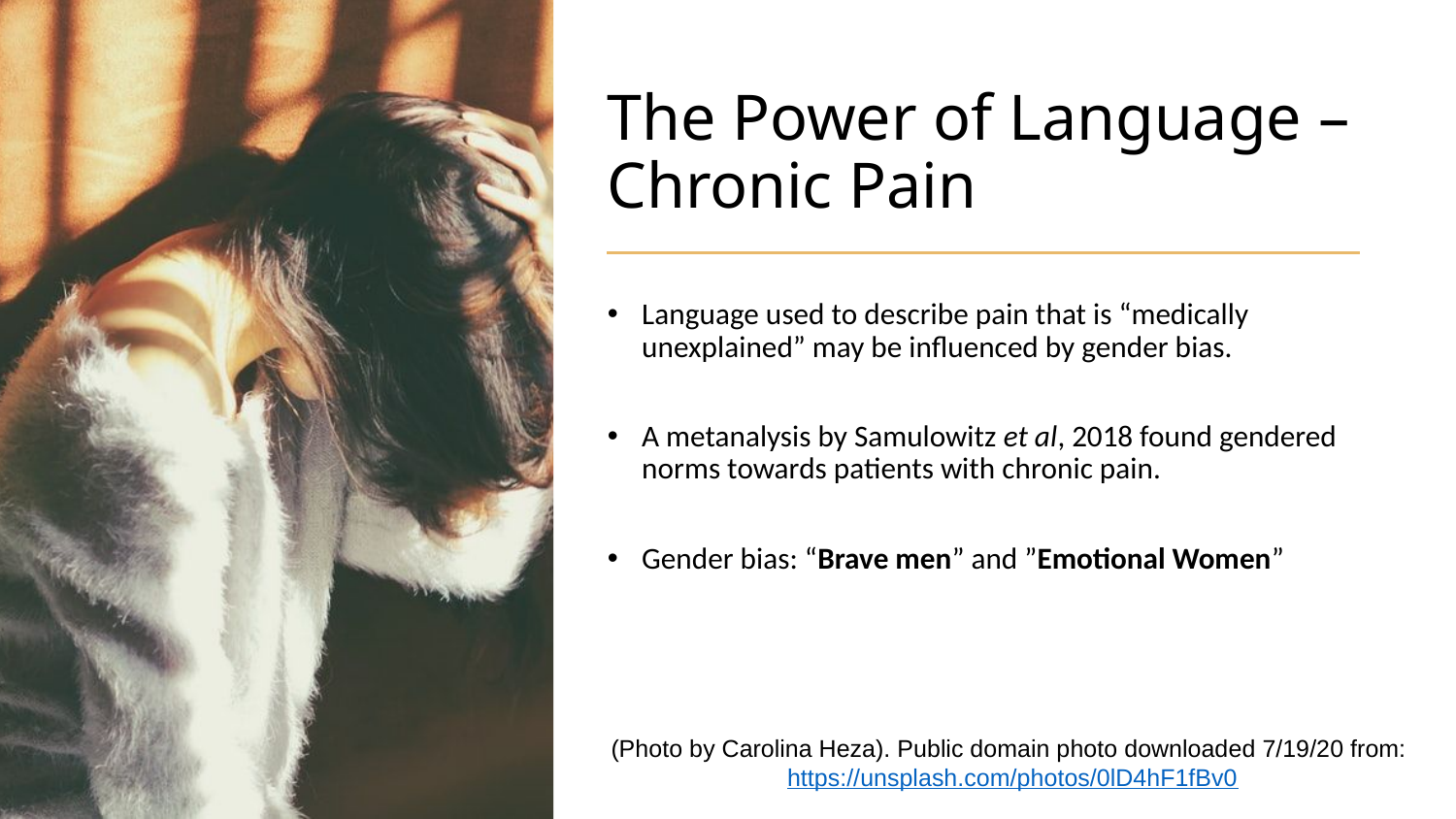

# The Power of Language – Chronic Pain
Language used to describe pain that is “medically unexplained” may be influenced by gender bias.
A metanalysis by Samulowitz et al, 2018 found gendered norms towards patients with chronic pain.
Gender bias: “Brave men” and ”Emotional Women”
(Photo by Carolina Heza). Public domain photo downloaded 7/19/20 from: https://unsplash.com/photos/0lD4hF1fBv0

## Slide 18
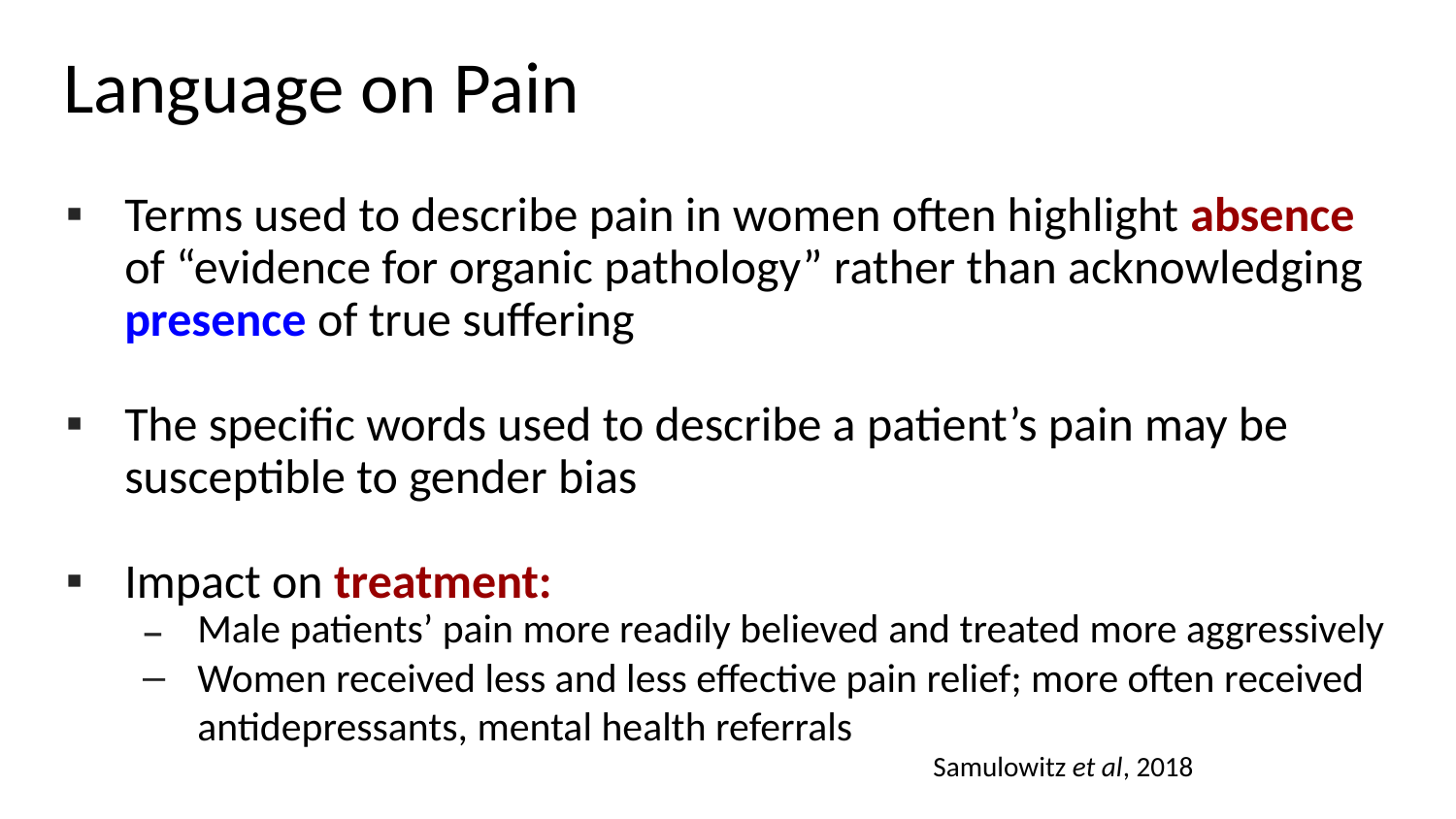

# Language on Pain
Terms used to describe pain in women often highlight absence of “evidence for organic pathology” rather than acknowledging presence of true suffering
The specific words used to describe a patient’s pain may be susceptible to gender bias
Impact on treatment:
Male patients’ pain more readily believed and treated more aggressively
Women received less and less effective pain relief; more often received antidepressants, mental health referrals
Samulowitz et al, 2018

## Slide 19
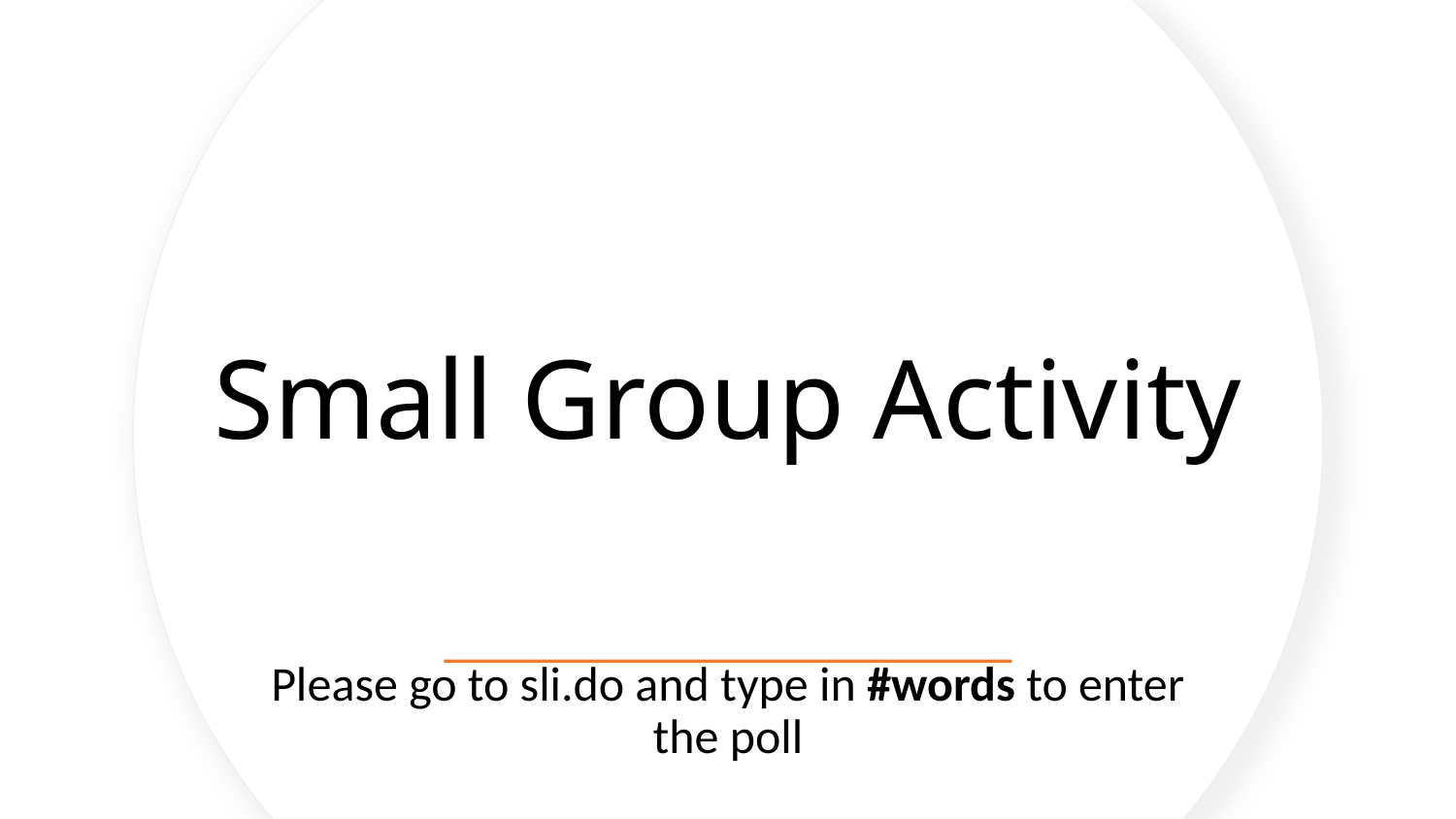

# Small Group Activity
Please go to sli.do and type in #words to enter the poll

## Slide 20
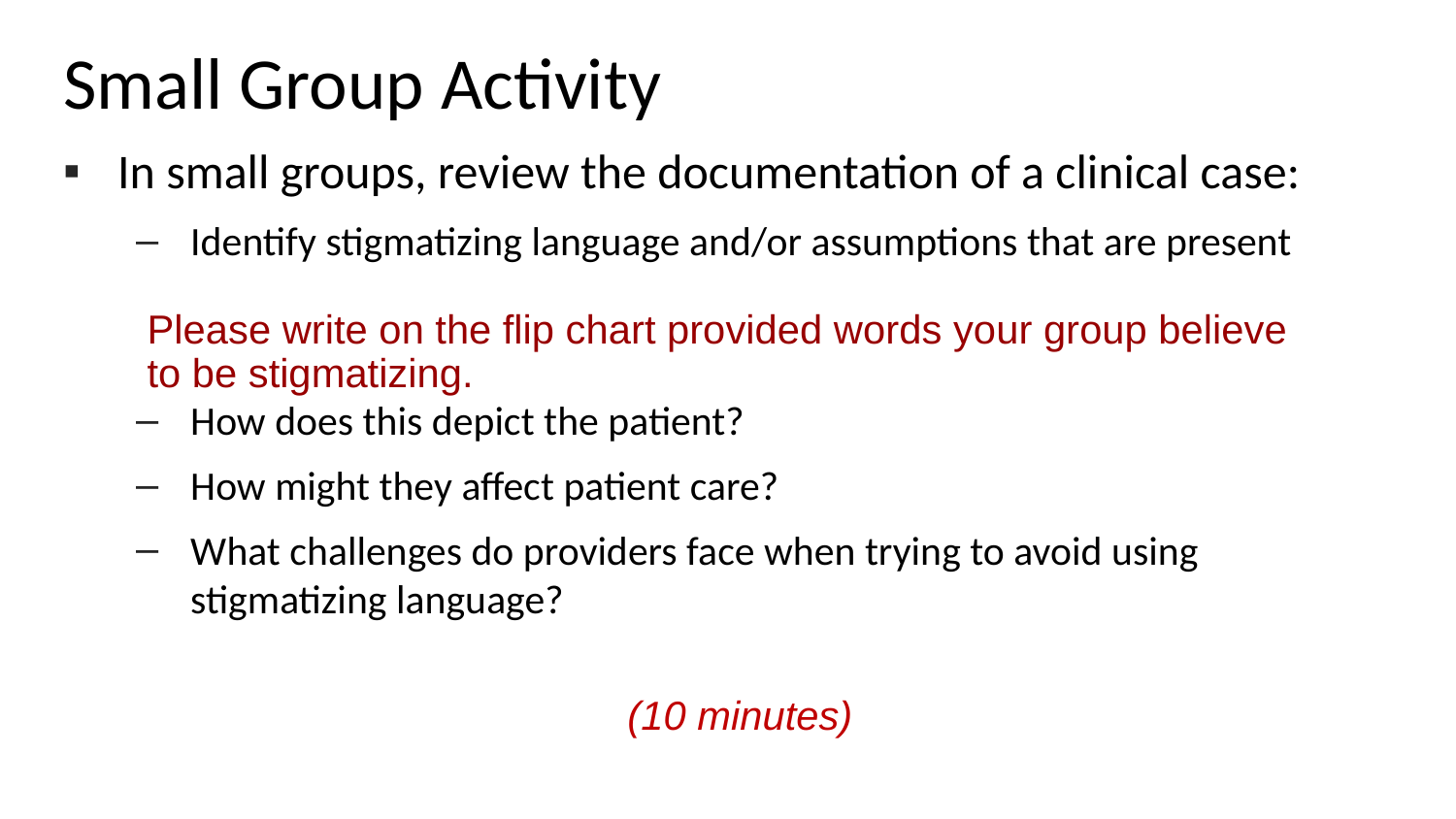

# Small Group Activity
In small groups, review the documentation of a clinical case:
Identify stigmatizing language and/or assumptions that are present
How does this depict the patient?
How might they affect patient care?
What challenges do providers face when trying to avoid using stigmatizing language?
Please write on the flip chart provided words your group believe to be stigmatizing.
(10 minutes)

## Slide 21
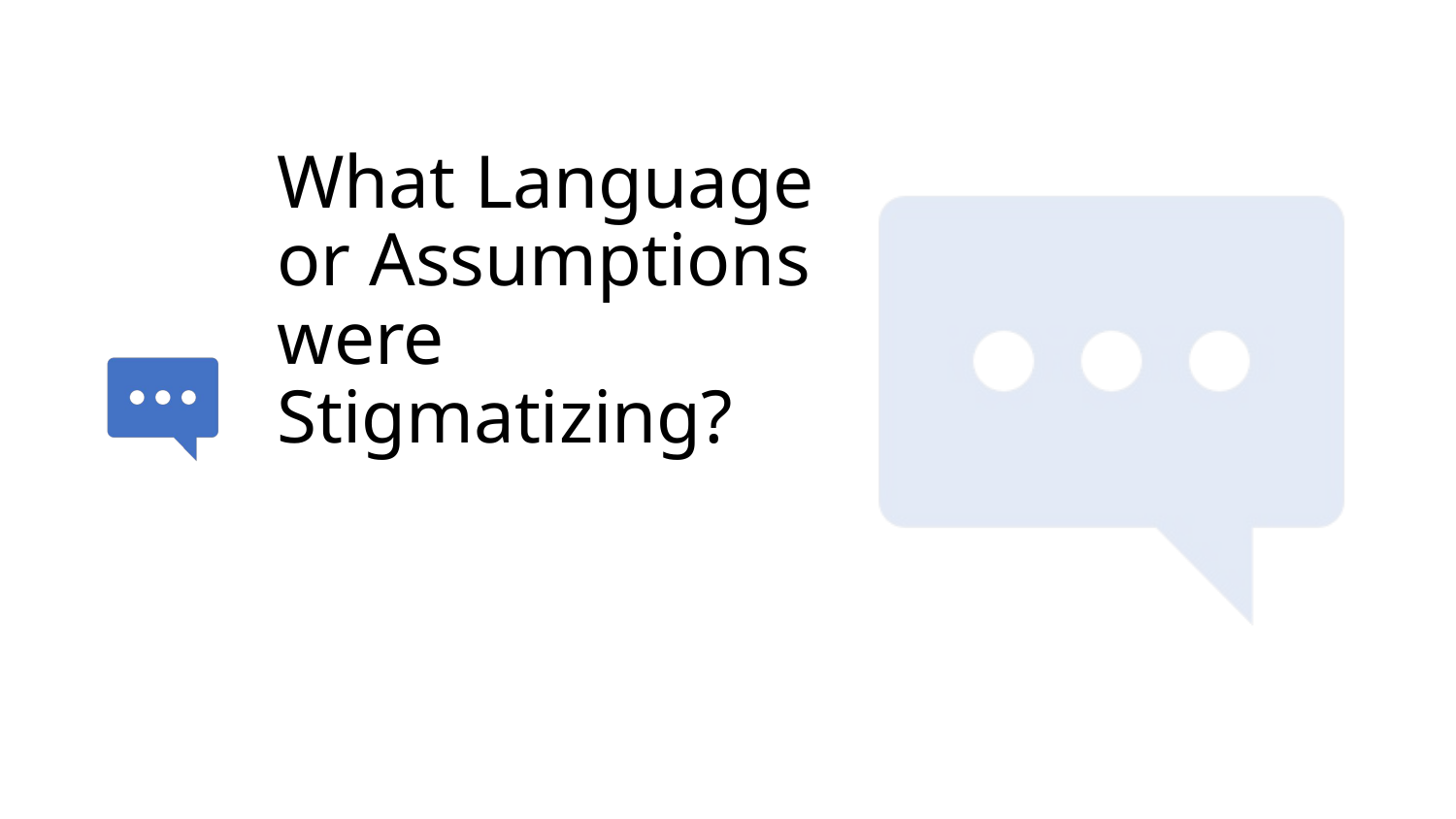

# What Language or Assumptions were Stigmatizing?

## Slide 22
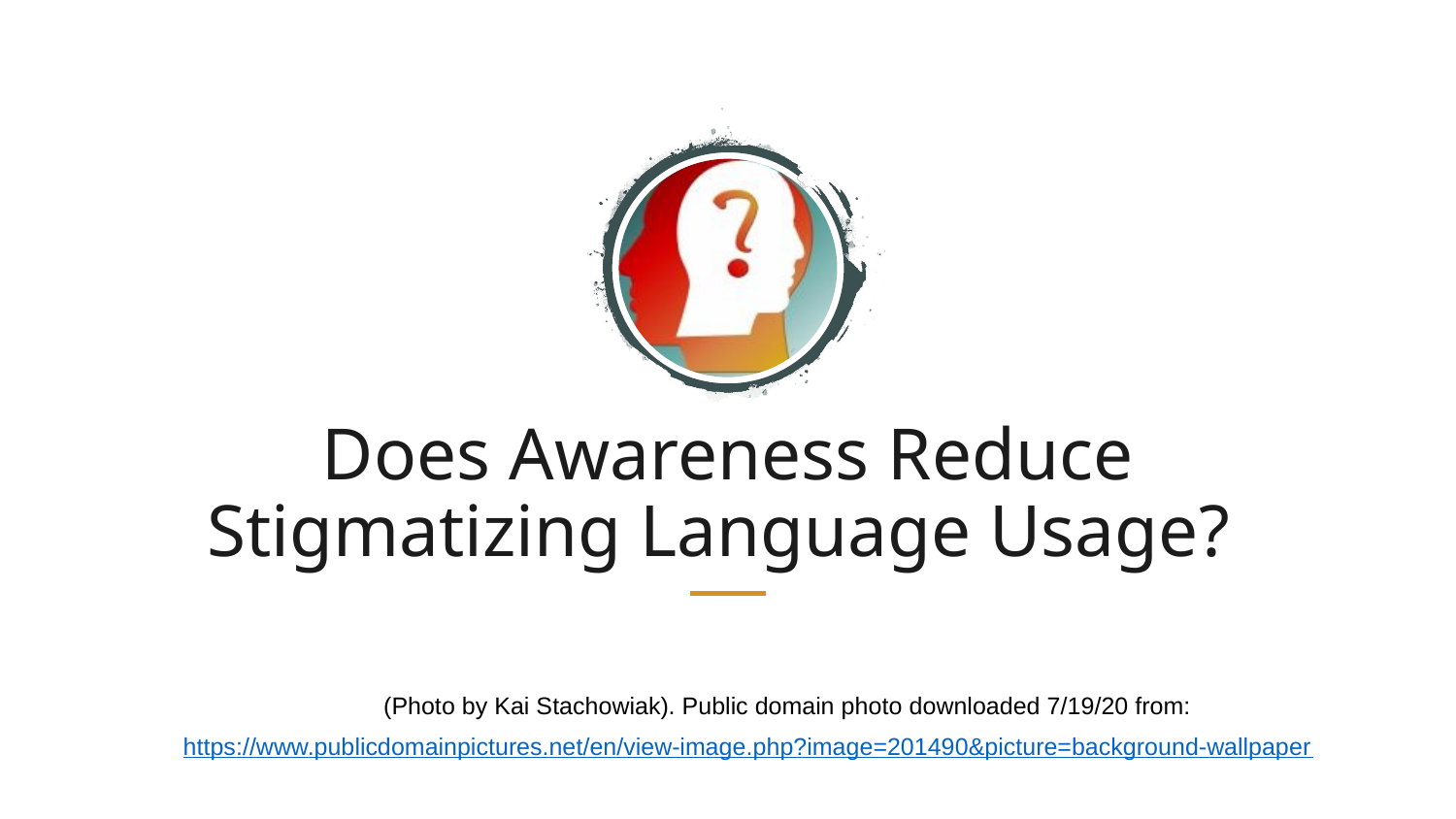

# Does Awareness Reduce Stigmatizing Language Usage?
(Photo by Kai Stachowiak). Public domain photo downloaded 7/19/20 from:
https://www.publicdomainpictures.net/en/view-image.php?image=201490&picture=background-wallpaper

## Slide 23
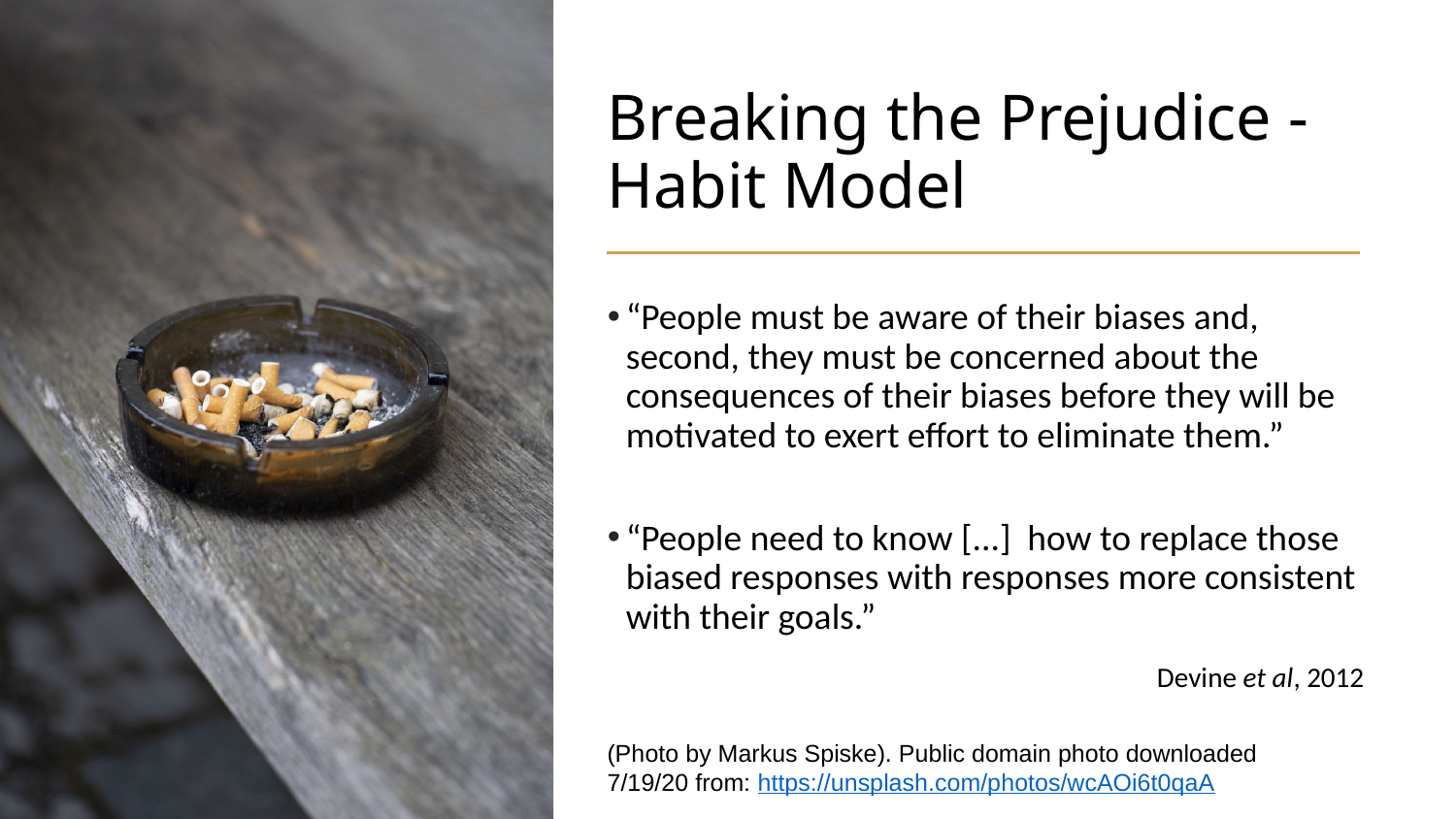

# Breaking the Prejudice - Habit Model
“People must be aware of their biases and, second, they must be concerned about the consequences of their biases before they will be motivated to exert effort to eliminate them.”
“People need to know [...] how to replace those biased responses with responses more consistent with their goals.”
Devine et al, 2012
(Photo by Markus Spiske). Public domain photo downloaded 7/19/20 from: https://unsplash.com/photos/wcAOi6t0qaA

## Slide 24
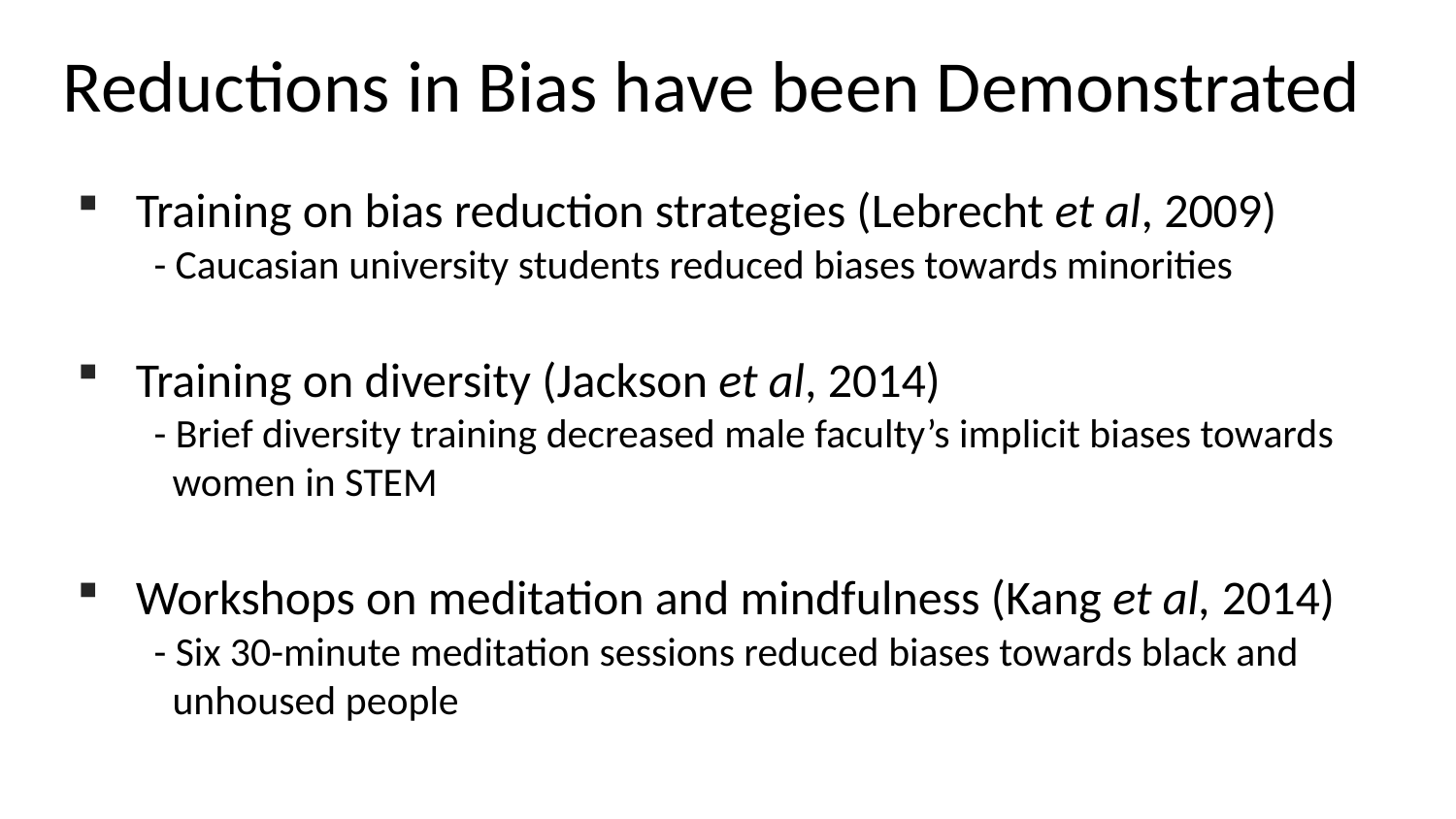

# Reductions in Bias have been Demonstrated
Training on bias reduction strategies (Lebrecht et al, 2009)
- Caucasian university students reduced biases towards minorities
Training on diversity (Jackson et al, 2014)
- Brief diversity training decreased male faculty’s implicit biases towards women in STEM
Workshops on meditation and mindfulness (Kang et al, 2014)
- Six 30-minute meditation sessions reduced biases towards black and unhoused people

## Slide 25
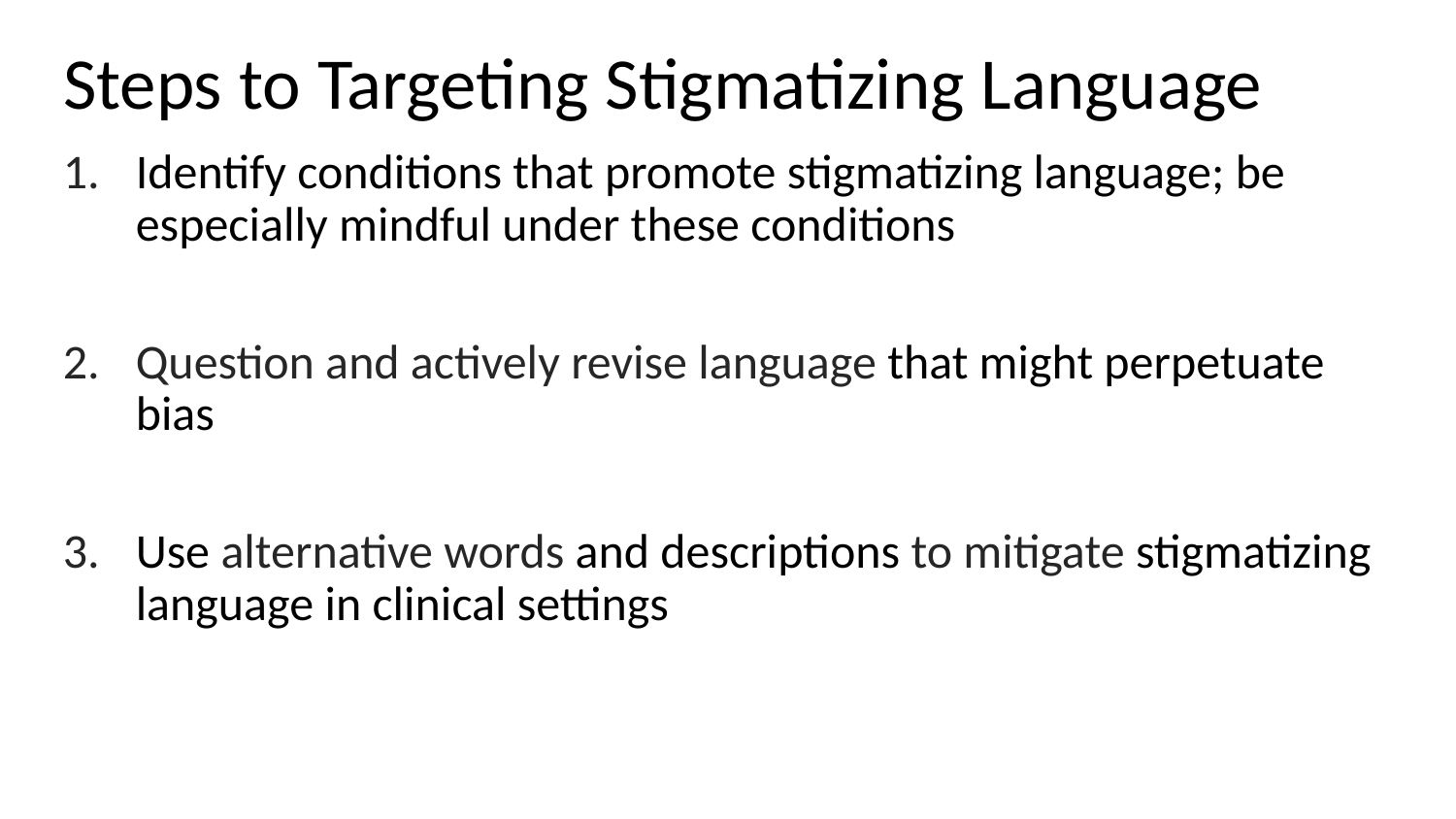

# Steps to Targeting Stigmatizing Language
Identify conditions that promote stigmatizing language; be especially mindful under these conditions
Question and actively revise language that might perpetuate bias
Use alternative words and descriptions to mitigate stigmatizing language in clinical settings

## Slide 26
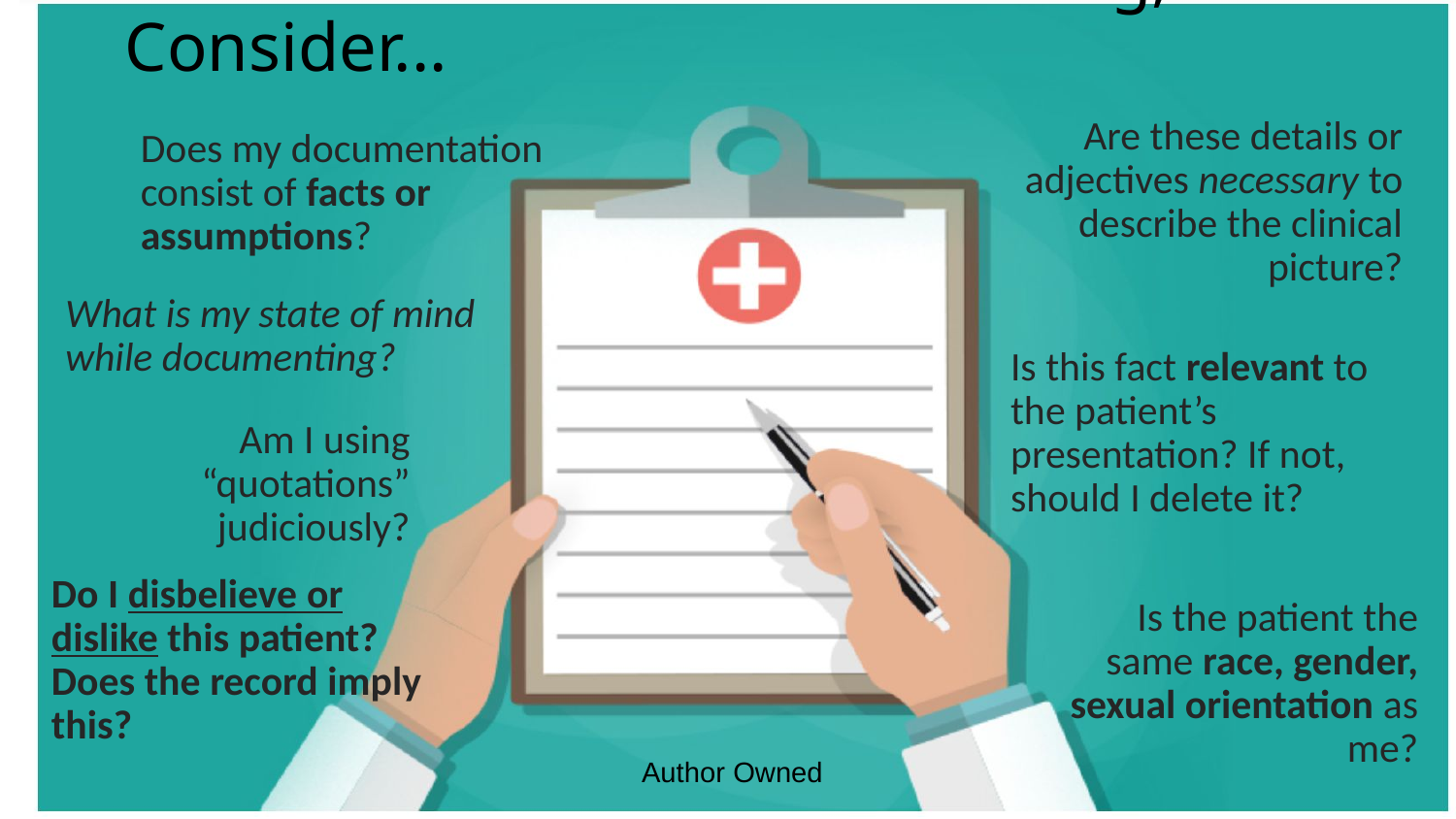

# Reflection: Before Documenting, Consider...
Are these details or adjectives necessary to describe the clinical picture?
Does my documentation consist of facts or assumptions?
What is my state of mind while documenting?
Is this fact relevant to the patient’s presentation? If not, should I delete it?
Am I using “quotations” judiciously?
Do I disbelieve or dislike this patient? Does the record imply this?
Is the patient the same race, gender, sexual orientation as me?
Author Owned

## Slide 27
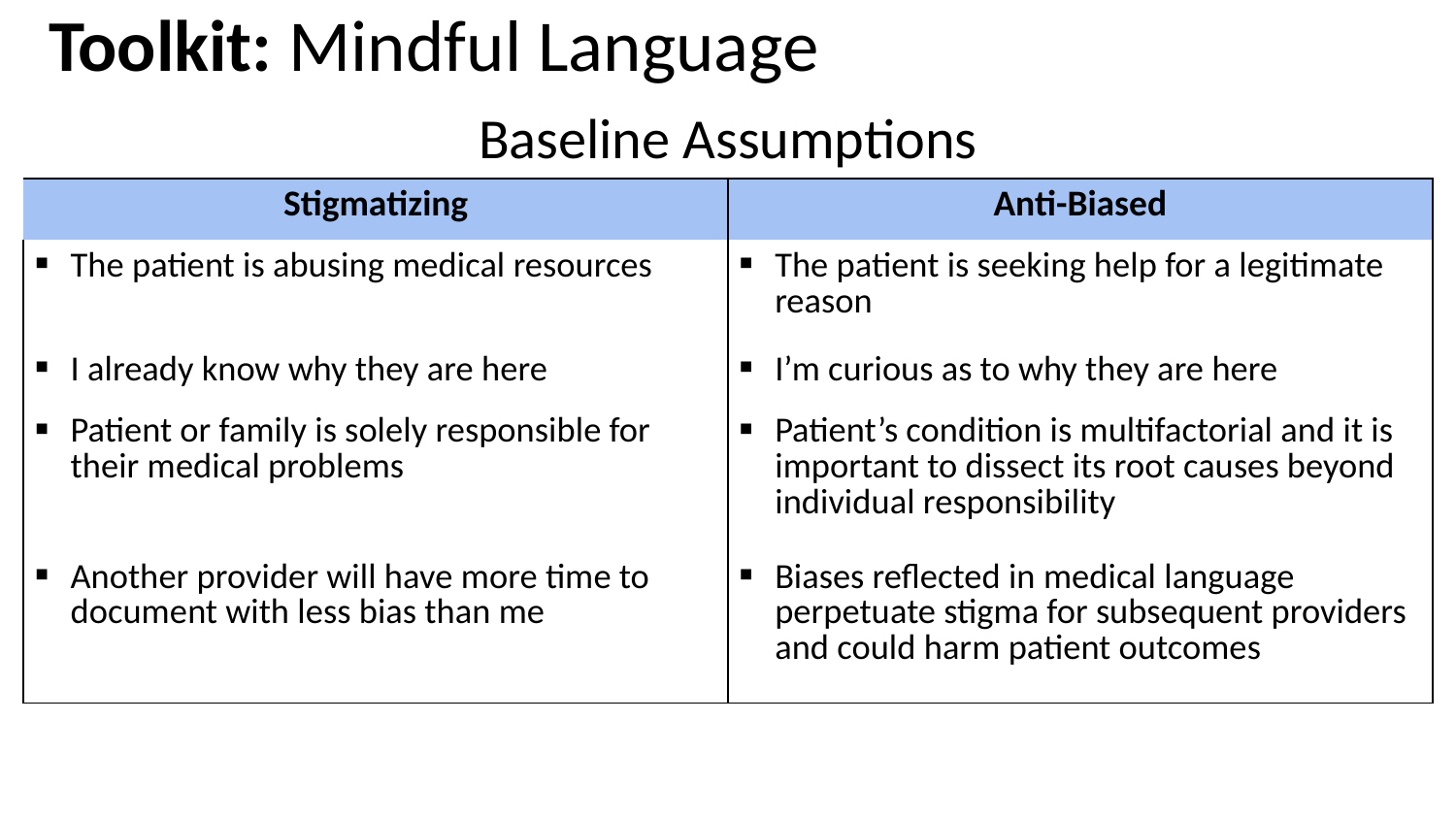

# Toolkit: Mindful Language
Baseline Assumptions
| Stigmatizing | Anti-Biased |
| --- | --- |
| The patient is abusing medical resources | The patient is seeking help for a legitimate reason |
| I already know why they are here | I’m curious as to why they are here |
| Patient or family is solely responsible for their medical problems | Patient’s condition is multifactorial and it is important to dissect its root causes beyond individual responsibility |
| Another provider will have more time to document with less bias than me | Biases reflected in medical language perpetuate stigma for subsequent providers and could harm patient outcomes |

## Slide 28
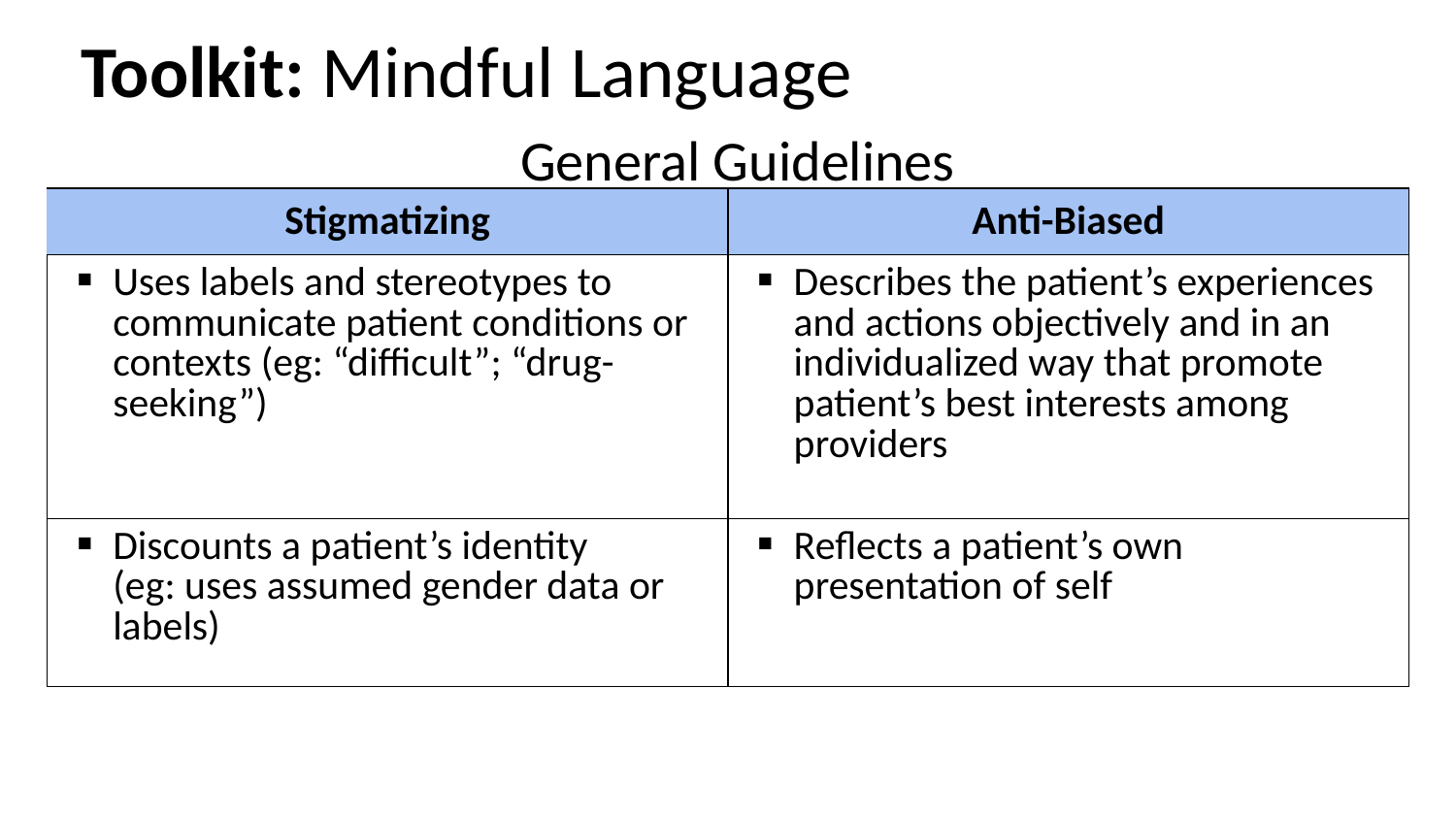

# Toolkit: Mindful Language
General Guidelines
| Stigmatizing | Anti-Biased |
| --- | --- |
| Uses labels and stereotypes to communicate patient conditions or contexts (eg: “difficult”; “drug-seeking”) | Describes the patient’s experiences and actions objectively and in an individualized way that promote patient’s best interests among providers |
| Discounts a patient’s identity (eg: uses assumed gender data or labels) | Reflects a patient’s own presentation of self |

## Slide 29
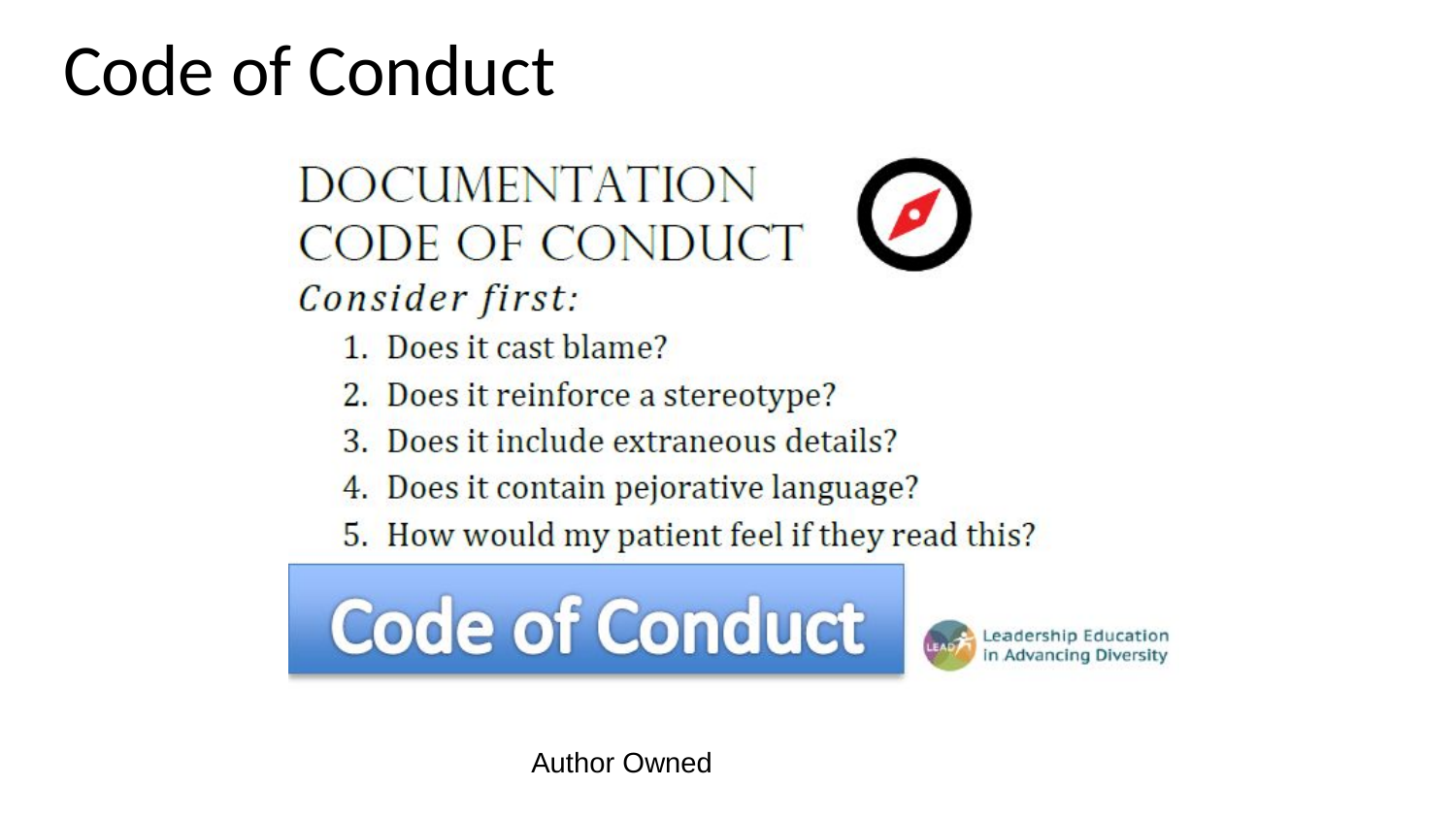

# Code of Conduct
Author Owned

## Slide 30
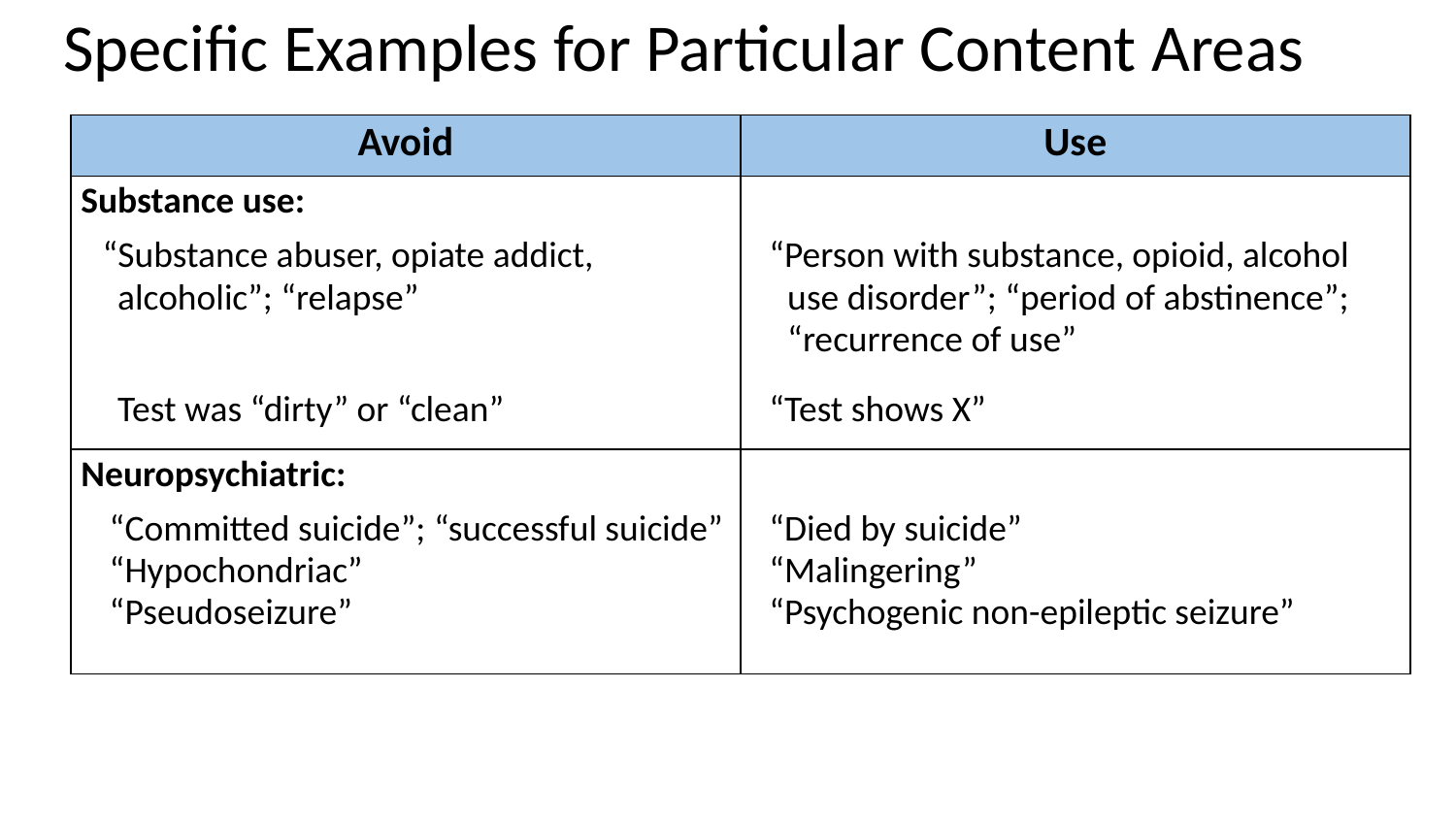

# Specific Examples for Particular Content Areas
| Avoid | Use |
| --- | --- |
| Substance use: “Substance abuser, opiate addict, alcoholic”; “relapse” | “Person with substance, opioid, alcohol use disorder”; “period of abstinence”; “recurrence of use” |
| Test was “dirty” or “clean” | “Test shows X” |
| Neuropsychiatric: “Committed suicide”; “successful suicide” “Hypochondriac” “Pseudoseizure” | “Died by suicide” “Malingering” “Psychogenic non-epileptic seizure” |

## Slide 31
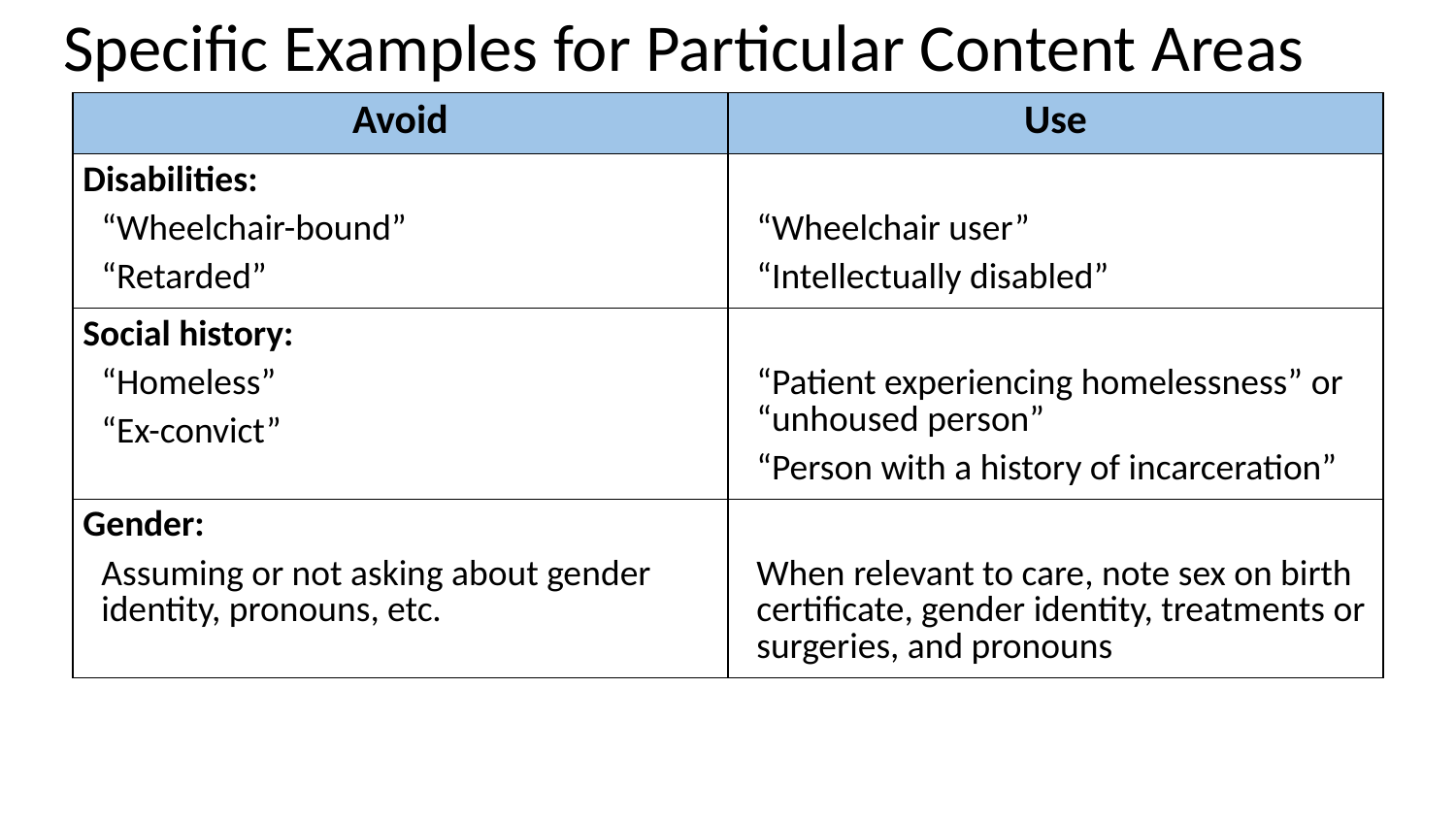

# Specific Examples for Particular Content Areas
| Avoid | Use |
| --- | --- |
| Disabilities: “Wheelchair-bound” “Retarded” | “Wheelchair user” “Intellectually disabled” |
| Social history: “Homeless” “Ex-convict” | “Patient experiencing homelessness” or “unhoused person” “Person with a history of incarceration” |
| Gender: Assuming or not asking about gender identity, pronouns, etc. | When relevant to care, note sex on birth certificate, gender identity, treatments or surgeries, and pronouns |

## Slide 32
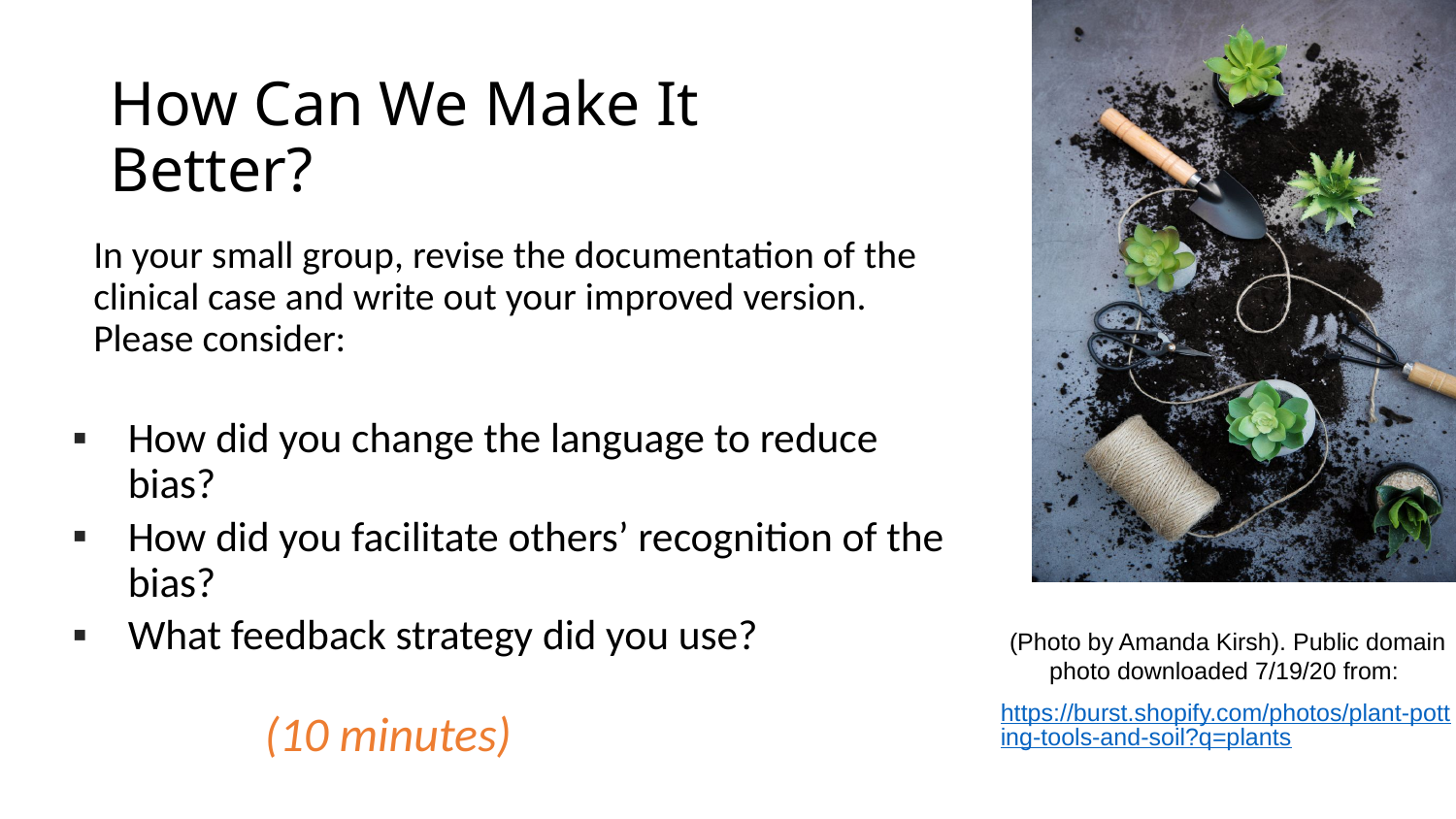

# How Can We Make It Better?
In your small group, revise the documentation of the clinical case and write out your improved version. Please consider:
How did you change the language to reduce bias?
How did you facilitate others’ recognition of the bias?
What feedback strategy did you use?
(Photo by Amanda Kirsh). Public domain photo downloaded 7/19/20 from:
https://burst.shopify.com/photos/plant-potting-tools-and-soil?q=plants
(10 minutes)

## Slide 33
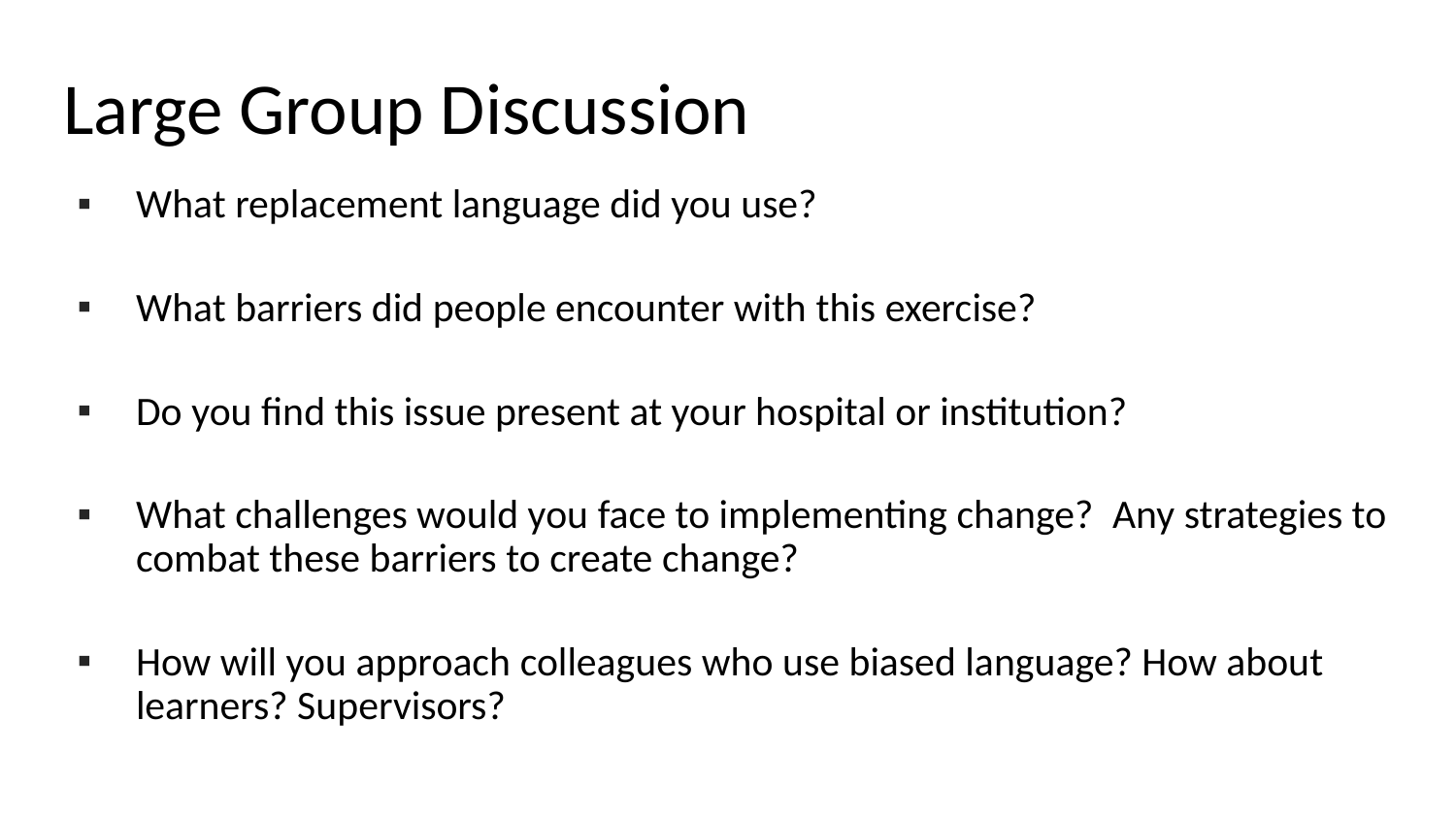

# Large Group Discussion
What replacement language did you use?
What barriers did people encounter with this exercise?
Do you find this issue present at your hospital or institution?
What challenges would you face to implementing change?  Any strategies to combat these barriers to create change?
How will you approach colleagues who use biased language? How about learners? Supervisors?

## Slide 34
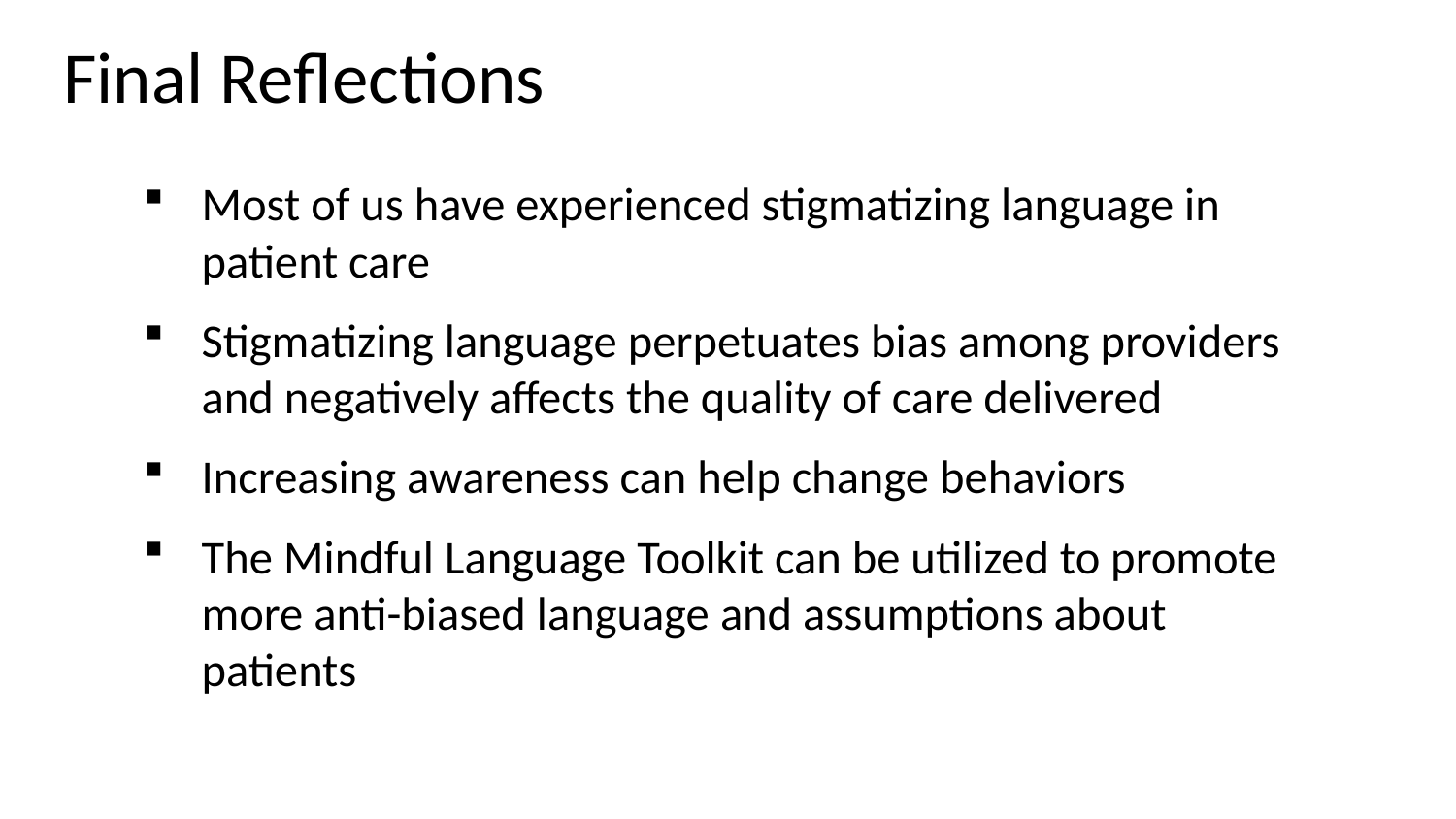

# Final Reflections
Most of us have experienced stigmatizing language in patient care
Stigmatizing language perpetuates bias among providers and negatively affects the quality of care delivered
Increasing awareness can help change behaviors
The Mindful Language Toolkit can be utilized to promote more anti-biased language and assumptions about patients

## Slide 35
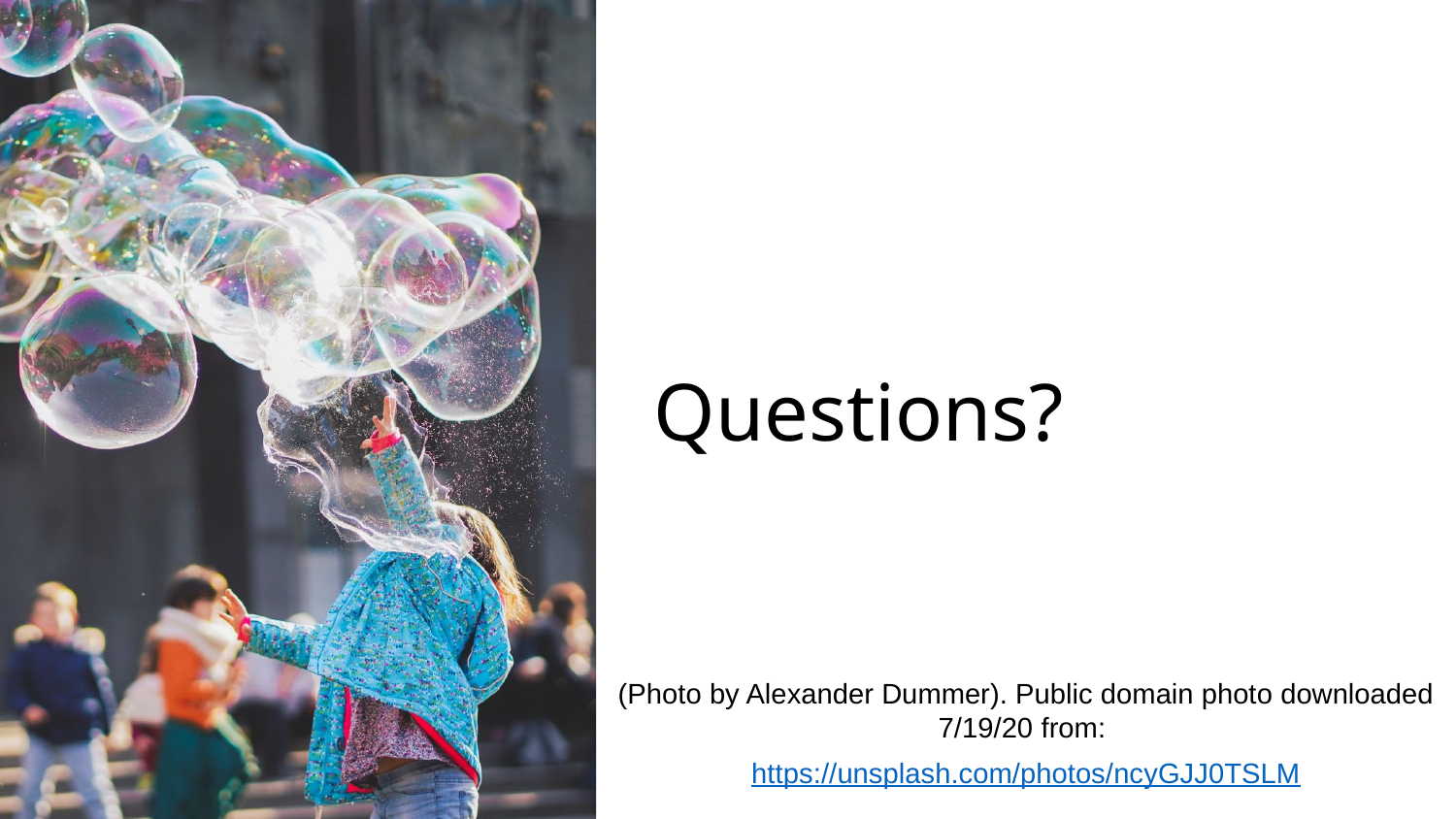

# Questions?
(Photo by Alexander Dummer). Public domain photo downloaded 7/19/20 from:
https://unsplash.com/photos/ncyGJJ0TSLM

## Slide 36
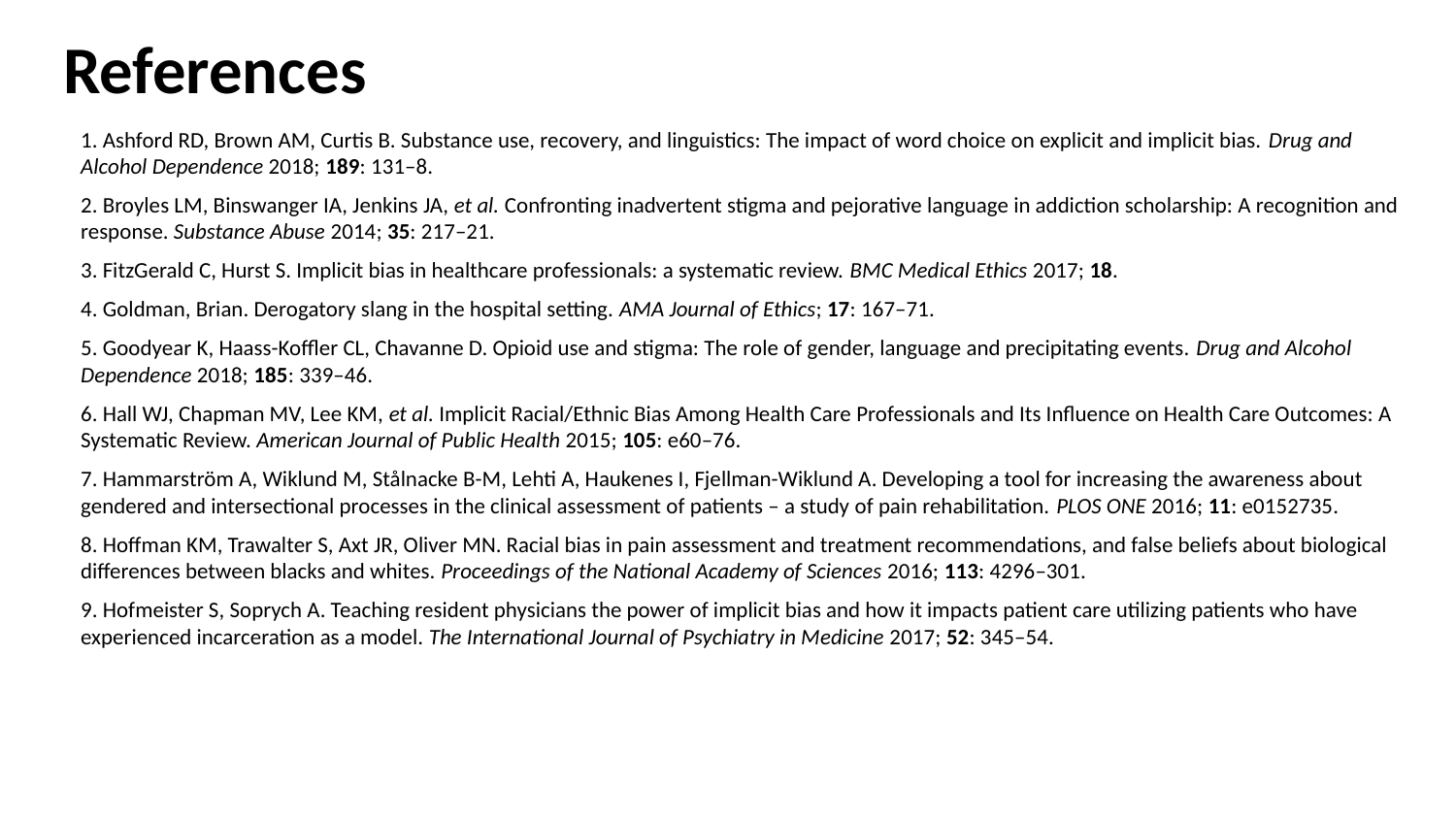

# References
1. Ashford RD, Brown AM, Curtis B. Substance use, recovery, and linguistics: The impact of word choice on explicit and implicit bias. Drug and Alcohol Dependence 2018; 189: 131–8.
2. Broyles LM, Binswanger IA, Jenkins JA, et al. Confronting inadvertent stigma and pejorative language in addiction scholarship: A recognition and response. Substance Abuse 2014; 35: 217–21.
3. FitzGerald C, Hurst S. Implicit bias in healthcare professionals: a systematic review. BMC Medical Ethics 2017; 18.
4. Goldman, Brian. Derogatory slang in the hospital setting. AMA Journal of Ethics; 17: 167–71.
5. Goodyear K, Haass-Koffler CL, Chavanne D. Opioid use and stigma: The role of gender, language and precipitating events. Drug and Alcohol Dependence 2018; 185: 339–46.
6. Hall WJ, Chapman MV, Lee KM, et al. Implicit Racial/Ethnic Bias Among Health Care Professionals and Its Influence on Health Care Outcomes: A Systematic Review. American Journal of Public Health 2015; 105: e60–76.
7. Hammarström A, Wiklund M, Stålnacke B-M, Lehti A, Haukenes I, Fjellman-Wiklund A. Developing a tool for increasing the awareness about gendered and intersectional processes in the clinical assessment of patients – a study of pain rehabilitation. PLOS ONE 2016; 11: e0152735.
8. Hoffman KM, Trawalter S, Axt JR, Oliver MN. Racial bias in pain assessment and treatment recommendations, and false beliefs about biological differences between blacks and whites. Proceedings of the National Academy of Sciences 2016; 113: 4296–301.
9. Hofmeister S, Soprych A. Teaching resident physicians the power of implicit bias and how it impacts patient care utilizing patients who have experienced incarceration as a model. The International Journal of Psychiatry in Medicine 2017; 52: 345–54.

## Slide 37
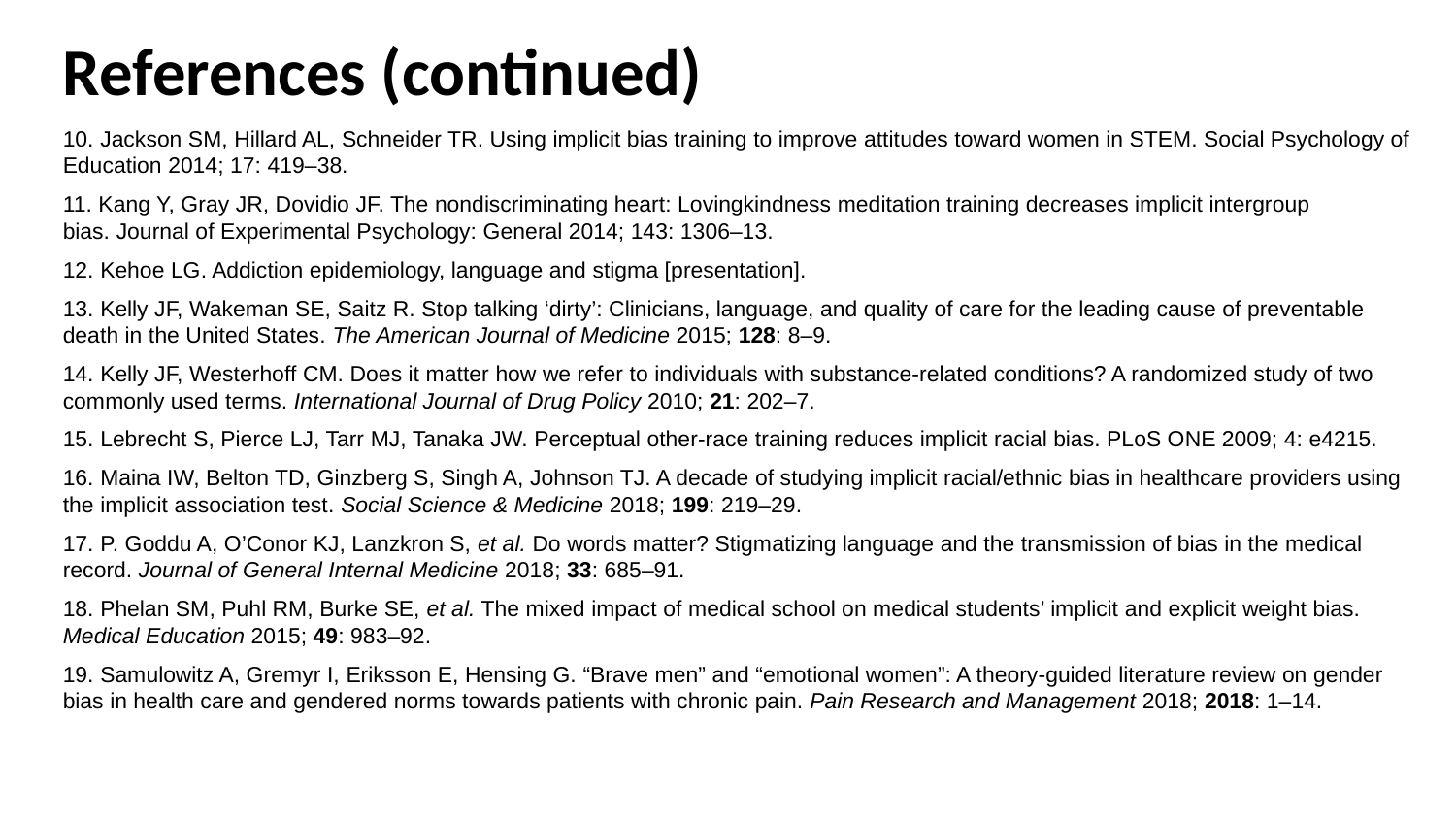

# References (continued)
10. Jackson SM, Hillard AL, Schneider TR. Using implicit bias training to improve attitudes toward women in STEM. Social Psychology of Education 2014; 17: 419–38.
11. Kang Y, Gray JR, Dovidio JF. The nondiscriminating heart: Lovingkindness meditation training decreases implicit intergroup bias. Journal of Experimental Psychology: General 2014; 143: 1306–13.
12. Kehoe LG. Addiction epidemiology, language and stigma [presentation].
13. Kelly JF, Wakeman SE, Saitz R. Stop talking ‘dirty’: Clinicians, language, and quality of care for the leading cause of preventable death in the United States. The American Journal of Medicine 2015; 128: 8–9.
14. Kelly JF, Westerhoff CM. Does it matter how we refer to individuals with substance-related conditions? A randomized study of two commonly used terms. International Journal of Drug Policy 2010; 21: 202–7.
15. Lebrecht S, Pierce LJ, Tarr MJ, Tanaka JW. Perceptual other-race training reduces implicit racial bias. PLoS ONE 2009; 4: e4215.
16. Maina IW, Belton TD, Ginzberg S, Singh A, Johnson TJ. A decade of studying implicit racial/ethnic bias in healthcare providers using the implicit association test. Social Science & Medicine 2018; 199: 219–29.
17. P. Goddu A, O’Conor KJ, Lanzkron S, et al. Do words matter? Stigmatizing language and the transmission of bias in the medical record. Journal of General Internal Medicine 2018; 33: 685–91.
18. Phelan SM, Puhl RM, Burke SE, et al. The mixed impact of medical school on medical students’ implicit and explicit weight bias. Medical Education 2015; 49: 983–92.
19. Samulowitz A, Gremyr I, Eriksson E, Hensing G. “Brave men” and “emotional women”: A theory-guided literature review on gender bias in health care and gendered norms towards patients with chronic pain. Pain Research and Management 2018; 2018: 1–14.

## Slide 38
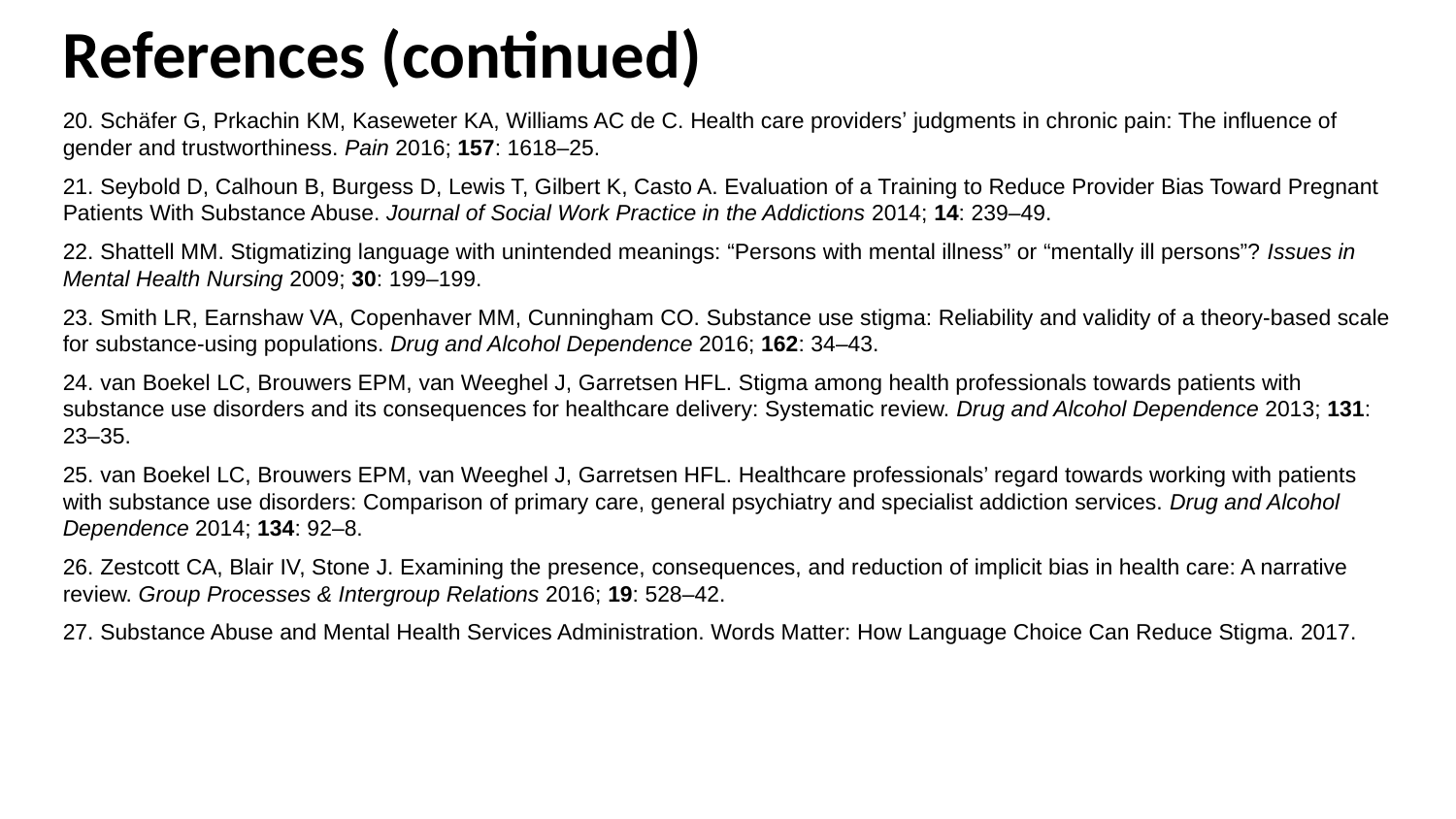

# References (continued)
20. Schäfer G, Prkachin KM, Kaseweter KA, Williams AC de C. Health care providersʼ judgments in chronic pain: The influence of gender and trustworthiness. Pain 2016; 157: 1618–25.
21. Seybold D, Calhoun B, Burgess D, Lewis T, Gilbert K, Casto A. Evaluation of a Training to Reduce Provider Bias Toward Pregnant Patients With Substance Abuse. Journal of Social Work Practice in the Addictions 2014; 14: 239–49.
22. Shattell MM. Stigmatizing language with unintended meanings: “Persons with mental illness” or “mentally ill persons”? Issues in Mental Health Nursing 2009; 30: 199–199.
23. Smith LR, Earnshaw VA, Copenhaver MM, Cunningham CO. Substance use stigma: Reliability and validity of a theory-based scale for substance-using populations. Drug and Alcohol Dependence 2016; 162: 34–43.
24. van Boekel LC, Brouwers EPM, van Weeghel J, Garretsen HFL. Stigma among health professionals towards patients with substance use disorders and its consequences for healthcare delivery: Systematic review. Drug and Alcohol Dependence 2013; 131: 23–35.
25. van Boekel LC, Brouwers EPM, van Weeghel J, Garretsen HFL. Healthcare professionals’ regard towards working with patients with substance use disorders: Comparison of primary care, general psychiatry and specialist addiction services. Drug and Alcohol Dependence 2014; 134: 92–8.
26. Zestcott CA, Blair IV, Stone J. Examining the presence, consequences, and reduction of implicit bias in health care: A narrative review. Group Processes & Intergroup Relations 2016; 19: 528–42.
27. Substance Abuse and Mental Health Services Administration. Words Matter: How Language Choice Can Reduce Stigma. 2017.
