# Supplementary material for: Words Matter: An Antibias Workshop for Health Care Professionals to Reduce Stigmatizing Language
Source: MedEdPORTAL. 2021 Mar 2;17:11115. doi: 10.15766/mep_2374-8265.11115 (PMC7970642; doi:10.15766/mep_2374-8265.11115)
Supplement: Supplementary file 1 — Facilitator's Guide.docxPowerPoint Presentation.pptxSign-out Skit.docxMindful Language Toolkit.docxClinical Cases.docxCourse Evaluation.docx [file mep_2374-8265.11115-s001.zip › C. Sign-Out Skit.docx]

**Appendix C: Sign-Out Skit**

**Resident 1:** Ok, are you ready for this sign out?

**Resident 2:** Sounds good.

**Resident 1:** My first patient is this 57-year old frequent flyer, homeless, alcoholic, substance abuser, though last urine toxicology was clean, with history of type 2 diabetes, who presented to the ED for bilateral forearm pain to request a note for work. I have not actually got a chance to speak to him, but I have the admission H&P.

He was found to be altered and diaphoretic in the waiting room, glucose in the 30s. He received D50 boluses and was started on D10 drip. He is a very poor historian, but claims he is supposed to be on glargine 30 units at night and lispro 10 units with completed random administration 1-2 times per day.

He has not checked his finger stick for 2 weeks, because he says this is annoying to him, and he takes his lispro whenever he feels like he should. Before he came in, he took his glargine, then lispro 15 units 30 min later for no clear reason. I do not believe he is suicidal. His reason is that he knew he was coming to the hospital and knew he would get his sugar checked and wanted his numbers to be low.

Other than eating 2 donuts in the morning, he has not eaten any food since, he already wanted to leave AMA, but was convinced to stay.

**Resident 2:** So this guy basically had it coming. Does he even know what insulin is?

**Resident 1:** Well he has been seen by the diabetic educator. I can say at least non-compliant.

The second patient is an 18-year-old Hispanic patient who is morbidly obese and borderline who is presenting with “10/10” abdominal pain. She is very anxious, and her mother is at bedside, who is a little crazy.

Last note I saw was to address her weight and to try lifestyle management, and the patient claims she has been trying to incorporate more fruits/vegetables into her diet in addition to walking more. Her diet consists mostly of McDonald’s and soda for lunch and dinner. She frequently misses breakfast. She is not participating in any exercise program, and is too anxious to discuss procedural interventions, really seems uninterested in discussing the health consequences of her obesity.

She is here for generalized abdominal pain, which does not seem to be new. She claims she has also been nauseated with decreased appetite and questionable weight loss.

**Resident 2:** What do you think is causing her pain?

**Resident 1:** Well her labs are still pending, so I don’t really know, but she is most likely pain seeking given that she came in asking for hydromorphone, which she has received in the past.

**Resident 2:** And her mom?

**Resident 1:** Asks a lot of questions and is overbearing, but medically illiterate, so I would probably try to avoid her room tonight if you can.
