# Supplementary material for: Words Matter: An Antibias Workshop for Health Care Professionals to Reduce Stigmatizing Language
Source: MedEdPORTAL. 2021 Mar 2;17:11115. doi: 10.15766/mep_2374-8265.11115 (PMC7970642; doi:10.15766/mep_2374-8265.11115)
Supplement: Supplementary file 1 — Facilitator's Guide.docxPowerPoint Presentation.pptxSign-out Skit.docxMindful Language Toolkit.docxClinical Cases.docxCourse Evaluation.docx [file mep_2374-8265.11115-s001.zip › D. Mindful Language Toolkit.docx]

Appendix D: Mindful  Language Toolkit

*Important Questions to Consider*

 
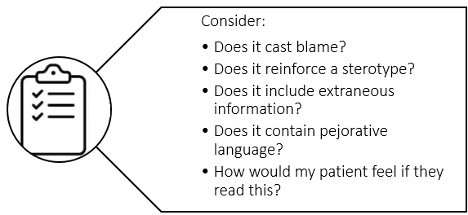


Author Owned

*Attitudes that communicate bias or neutrality in medical documentation:*

| **Stigmatizing** | **Neutral** |
| --- | --- |
| Patient is abusing medical resources | Patient is here to seek help |
| Using labels and stereotypes to communicate patient conditions or contexts (e.g.: “difficult”; “drug-seeking”) | Objective, individualized descriptions of the patient’s experiences and actions promote patients’ best interests among providers |
| Documentation discounts patient’s identity, uses assumed gender data or avoids topic altogether | Documentation reflects patient’s presentation of self |
| Patient is solely responsible for their medical problems | Patient’s condition is multifactorial, and it is important to dissect its root causes beyond individual responsibility |
| Another provider will have more time to document with less bias than me | Biases reflected in medical documentation/language perpetuate stigma for each subsequent provider |

*General tips*

| **Avoid** | **Use** |
| --- | --- |
| Making the patient their disease (e.g. “she is bipolar”; “sickler”; “substance abuser”) | Phrases like “He has X disorder” |
| Using unnecessary quotations | Patient’s exact words judiciously |
| Words that cast doubt on a patient's experience (i.e.: patient claims, believes…) | Neutral language that accurately relays history (i.e.: patient reports, states…) |
| Words which imply decreased agency for patients with disabilities (wheelchair bound versus wheelchair user) | Patients’ own descriptions of their disabilities |
| Be aware of words that might be perceived as racist, sexist, or stereotyped  “That’s retarded”  “She’s hysterical” | Patient’s subjective experience of their illness |
| Extraneous details that do not impact the condition for which the patient is presenting (e.g. incarceration history, race) | Using pertinent details only |
| Writing in a way that would be problematic for a patient or family member to read | Writing transparently, clearly |
| Using pejorative, dated terms for disease (“pseudoseizure,” “vegetable,” “retarded”) | Appropriate disease terminology (“PNES,” “minimally conscious state,” “intellectual disability”) |

*Special considerations for specific content areas*

| **Avoid** | **Use** |
| --- | --- |
| **Substance use:**  “Substance abuser, opiate addict, alcoholic”    Test was “dirty” or “clean” | “Person with substance, opioid, or alcohol use disorder”; “period of abstinence”    “Your test shows X” |
| **Mental health:**  “S/he’s bipolar”; “crazy”; “borderline”  “Committed suicide”; “successful suicide” | “SF has a known diagnosis of bipolar I disorder”  “Died by suicide” |
| **Disabilities:**  “Wheelchair-bound”  “Deaf and dumb” | “Wheelchair user”  “Hearing- and speech-impaired” |
| **Social history:**  “Homeless person”  “Ex-convict” | “Patient experiencing homelessness” or “unhoused person”—can specify chronic or acute  “Person with a history of incarceration” |
| **Gender:**  Assuming or not asking about gender identity, pronouns, etc. | When relevant to care, note sex on birth certificate, gender identity, treatments or surgeries, and pronouns |
