# Supplementary material for: Words Matter: An Antibias Workshop for Health Care Professionals to Reduce Stigmatizing Language
Source: MedEdPORTAL. 2021 Mar 2;17:11115. doi: 10.15766/mep_2374-8265.11115 (PMC7970642; doi:10.15766/mep_2374-8265.11115)
Supplement: Supplementary file 1 — Facilitator's Guide.docxPowerPoint Presentation.pptxSign-out Skit.docxMindful Language Toolkit.docxClinical Cases.docxCourse Evaluation.docx [file mep_2374-8265.11115-s001.zip › E. Clinical Cases.docx]

**Appendix E: Clinical Cases with Stigmatizing Language**

**Case 1: Pediatrics**

AJ is a 17-year-old girl with a history of complex childhood trauma, PTSD, borderline personality disorder, obesity, irritable bowel syndrome, Ehlers-Danlos syndrome with associated visceral hypersensitivity, and dysmotility, who is undergoing inpatient pain management for an acute pain flare. She is currently on total parenteral nutrition due to inability to tolerate food by mouth.

She is brought in by her mother, who is also seen as demanding and medically unsavvy. They are a bounce-back to the inpatient pediatrics service after a recent discharge and no-showing to post-hospitalization discharge follow-up outpatient appointment. Of note, patient has been a frequent flier to the ED and to multiple institutions seeking urgent narcotic and interventional inputs for similar symptoms.

Her current level of pain varies between 10/10 and 0/10, depending on the moment. AJ is an exceedingly unreliable historian—her descriptions of events and complaints of pain shift frequently with retelling; she is very hard to redirect. She is also attention-seeking; she contorts and moans in pain when in earshot of providers, but when she thinks she is alone, does not appear to be in any distress, sitting up in bed and talking loudly on the phone.

No objective or organic etiology has been found to date for her symptoms, despite several comprehensive work-ups. All labs and imaging have been completely normal. Patient’s mother and patient refuse to accept that no further diagnostic interventions are recommended and are demanding to see the most senior doctor in the hospital. They also demand to be cared for by more competent physicians, RNs, and consultants.

**Case 2: Adult Emergency Medicine**

WZ is a 26-year-old Spanish-speaking male who has antisocial personality disorder and is an alcoholic and polysubstance abuser, presenting to the emergency department for (now resolved) intermittent chest pain and shortness of breath. His most recent episode was earlier this morning and has resolved completely; he now appears completely comfortable and is repeatedly demanding food and work excuse notes, despite stating that he needs a full workup.

WZ claims that the chest pain began several weeks ago without any apparent inciting factors and he describes it as episodic. He reports that the pain lasts for 3-5 minutes, yet is “debilitating,” rating it as a “20 out of 10.” States that it often begins with a sensation “like choking.” No aggravating/alleviating factors. (He has tried Tylenol x 1 without improvement.) Reports no association with eating or positional changes. Reports no recent stressors.

Of note, patient is a frequent flyer to the ED (please see notes from prior ED encounters). During each visit, EKG, troponins, D-dimer, BMP, CBC have been wnl (though has had multiple dirty u tox results: + for cannabinoids, cocaine).

Endorses historical alcohol abuse and also admits to past drug abuse, though patient claims that he has been clean for 2-3 weeks. Lives at home with his single mother and several younger siblings, having dropped out of high school several years ago, with prior gang involvement and an incarceration history. Currently works part-time at a restaurant and is asking each provider for work excuse notes incessantly.

At this time, vitals and exam seem entirely benign.

**Revised Clinical Cases:** *These cases use what we consider to be anti-biased language. However, we do not expect that these cases are perfect. Please reflect on what aspects of these cases could be improved yet again.*

**Case 1 with suggested improvements: Pediatrics**

AJ is a 17-year-old adolescent (she/her/hers) with a history of complex childhood trauma, PTSD, borderline personality disorder, obesity, irritable bowel syndrome, Ehlers-Danlos syndrome with associated visceral hypersensitivity, and dysmotility, who is undergoing inpatient pain management for an acute pain flare, currently on total parenteral nutrition. She is currently on total parenteral nutrition due to inability to tolerate food by mouth.

She is brought in by her mother, who has low health literacy. She is readmitted after a recent discharge.

Of note, AJ has visited a number of EDs at multiple institutions seeking urgent narcotic and interventional inputs.

Her current level of pain varies between 10/10 and 0/10, depending on the moment. AJ’s descriptions of prior events and pain reports shift on occasion; at times, she is hard to redirect.

Labs and imaging have been unrevealing to date.

**Case 2 with suggested improvements: Emergency Medicine**

WZ is a 26-year-old Spanish-speaking male (he/him/his) with antisocial personality disorder, alcohol and polysubstance use disorder, presenting to the emergency department for (now resolved) intermittent chest pain and shortness of breath. His most recent episode was earlier this morning and has since resolved. He now appears comfortable.

WZ says that his chest pain began several weeks ago without any known inciting factors. He describes it as episodic, reporting that the pain lasts for 3-5 minutes and is debilitating, rating it as a 20 out of 10. States that it often begins with a sensation “like choking.” No known aggravating/alleviating factors. (He has tried Tylenol x 1 without improvement.) Does not report association with eating or positional changes. Does not endorse recent stressors.

WZ has presented to the ED for this pain a number of times. During each visit, EKG, troponins, D-dimer, BMP, CBC have been wnl; of note, u tox results have been positive for cannabinoids and cocaine.

WZ says he has not been using alcohol or drugs for the last 2-3 weeks. He lives at home with his mother and several younger siblings. Currently works part-time at a restaurant.

At this time, vitals and exam seem entirely benign.
