# Supplementary material for: Words Matter: An Antibias Workshop for Health Care Professionals to Reduce Stigmatizing Language
Source: MedEdPORTAL. 2021 Mar 2;17:11115. doi: 10.15766/mep_2374-8265.11115 (PMC7970642; doi:10.15766/mep_2374-8265.11115)
Supplement: Supplementary file 1 — Facilitator's Guide.docxPowerPoint Presentation.pptxSign-out Skit.docxMindful Language Toolkit.docxClinical Cases.docxCourse Evaluation.docx [file mep_2374-8265.11115-s001.zip › F. Course Evaluation.docx]

**Words Hurt: Identifying and Removing Stigmatizing Language from Clinical Settings**

EVALUATION FORM

**Profession: _______**

**Years in Practice:  ______**

**Workshop Objectives:**

1. Explain the impact of providers’ language biases on patient care
2. Describe strategies that can be used to mitigate providers’ language biases
3. Apply strategies from the *Mindful Language Toolkit* to address stigmatizing language

I am more aware of the impact of biased language on patient care:

Strongly disagree    Disagree      Neither agree/disagree      Agree          Strongly agree

I learned new strategies that I can use to identify and replace language biases:

Strongly disagree    Disagree      Neither agree/disagree      Agree          Strongly agree

I will apply the new anti-bias strategies I learned today to my clinical documentation:

Strongly disagree    Disagree      Neither agree/disagree      Agree          Strongly agree

I will  apply the new anti-bias strategies I learned today to how I discuss patients in the clinical environment:

Strongly disagree    Disagree      Neither agree/disagree      Agree          Strongly agree

The interprofessional participation of the presentation was valuable to the workshop:

Strongly disagree    Disagree      Neither agree/disagree      Agree          Strongly agree

 What two things will you do as a result of this workshop?

What do you see as potential barriers to applying what you have learned?

What did you like best about the workshop?

What can we improve about the workshop?
