# Supplementary material for: Words Matter: An Antibias Workshop for Health Care Professionals to Reduce Stigmatizing Language
Source: MedEdPORTAL. 2021 Mar 2;17:11115. doi: 10.15766/mep_2374-8265.11115 (PMC7970642; doi:10.15766/mep_2374-8265.11115)
Supplement: Supplementary file 1 — Facilitator's Guide.docxPowerPoint Presentation.pptxSign-out Skit.docxMindful Language Toolkit.docxClinical Cases.docxCourse Evaluation.docx [file mep_2374-8265.11115-s001.zip › _Educational_Summary_Report_11115.pdf]

MedEdPORTAL® | The AAMC Journal of Teaching and Learning Resources

Original Publication Open Access

Words Matter: An Antibias Workshop for Health Care Professionals to Reduce Stigmatizing Language

Julia Raney, MD*, Ria Pal, MD, Tiffany Lee, MD, Samuel Ricardo Saenz, MD, MPH, Devika Bhushan, MD, Peter Leahy, MD, Carrie Johnson, MBA, Cynthia Kapphahn, MD, MPH, Michael A. Gisondi, MD, Kim Hoang, MD

*Corresponding author: julia.raney4@gmail.com

Abstract

Introduction: Biased language influences health care providers' perceptions of patients, impacts their clinical care, and prevents vulnerable populations from seeking treatment. Training clinicians to systematically replace biased verbal and written language is an essential step to providing equitable care. **Methods:** We designed and implemented an interactive workshop to teach health care professionals a framework to identify and replace stigmatizing language in clinical practice. The workshop included a reflective exercise, role-play, brief didactic session, and case-based discussion. We developed the program for a broad target audience of providers and initially delivered it at three academic conferences. We used descriptive statistics to analyze Likert-style items on course evaluations and identified themes in open-text responses. **Results:** A total of 66 participants completed course evaluations; most believed the workshop met its objectives (4.8 out of 5.0) and strongly agreed that they would apply skills learned (4.8). Participants planned to incorporate reflection into their verbal and written language. Potential barriers to applying course content included perceived difficulty in changing entrenched practice habits, burnout, and fatigue. Suggestions for improvement included more time for group discussions and strategies to teach skills to colleagues. **Discussion:** Participants found the course material highly engaging and relevant to their clinical practice. Learners left the workshop feeling motivated to engage in more mindful word choice and to share key concepts with their colleagues.

Keywords
Health Disparities, Bias, Language, Communication Skills, Cultural Competence, Diversity, Inclusion, Health Equity, Professionalism, Case-Based Learning, Anti-racism

Educational Objectives

By the end of this activity, learners will be able to:

1. Explain the impact of providers' language biases on patient care.
2. Describe strategies that can be used to mitigate providers' language biases.
3. Apply strategies from the Mindful Language Toolkit to address stigmatizing language.

Introduction

There is increasing evidence that biased spoken and written language influences perceptions of patients and negatively affects treatment plans.[1-5] Goddu and colleagues' 2018 study exploring the impact of biased documentation found that medical students and residents randomized to read a clinical vignette using biased language to describe a patient with sickle cell disease were more likely to have a negative perception of the patient and less likely to recommend opioid analgesicss.[1] A similar pattern was found among mental health professionals who read a clinical vignette of a patient referred to as a substance abuser versus substance user; participants who read descriptions of a substance abuser were more likely to make character judgments and recommend punitive measures.[2] Biased language in the electronic health record may negatively influence future provider perceptions and patient care as well.

Patients with stigmatizing conditions such as diabetes, obesity, substance use disorder, and chronic pain are affected by the language of their health care providers.[6-11] Biased language can worsen feelings of shame, decreasing patients' motivations to complete their treatment plans or engage in treatment at all.[9,12] Therefore, biased language, likely due to assumptions made

Citation:
Raney J, Pal R, Lee T, et al. Words matter: an antibias workshop for health care professionals to reduce stigmatizing language. *MedEdPORTAL*. 2021;17:11115.
https://doi.org/10.15766/mep_2374-8265.11115

Copyright © 2021 Raney et al. This is an open-access publication distributed under the terms of the Creative Commons Attribution license.

from the environment and throughout training, plays a central role in perpetuating health care disparities. As medical records become increasingly accessible to patients and their families, the importance of unbiased documentation that supports patient agency is magnified.

MedEdPORTAL has several examples of workshops promoting antioppressive language use. For example, Mayfield and colleagues' LGBTQ-inclusive sexual history taking curriculum teaches the importance of using appropriate terms and gender-neutral language.[13] Stagno, Crapanzano, and Schwart's workshop for mental health audiences highlights the importance of using person-first language and removing judgmental words such as *denies* or *claims.*[14] However, there are no antibias workshops that provide a framework for identifying verbal and written bias in other clinical contexts.

Here, we describe the development of a language-based, antibias workshop and provide the materials necessary to present this training program. The course specifically addresses stigmatizing language in both written and verbal communication, including health records and clinical presentations, using a framework that reinforces antibias and antioppressive skills. The target audience is broad, including any provider, trainee, or staff member who works with patients in a clinical environment. We describe the power of stigmatizing language in medicine and situations in which clinicians often use biased language, as well as providing learners with tools to replace biased language.

Methods

This workshop was designed by participants and facilitators at the Stanford School of Medicine Leadership Education in Advancing Diversity (LEAD) program, including residents, fellows, program administrators, and teaching faculty.[15] Several members of this team had prior experience developing and facilitating antibias trainings. Three investigators were clinician educators with experience in workshop design and faculty development; three investigators were LEAD program instructors with content expertise in equity and inclusion; one trainee had prior experience developing and presenting an antibias workshop. Team members completed an extensive literature review of stigmatizing language in medicine to identify workshop content; search terms included the specific conditions of obesity, addiction, and chronic pain. We selected Sukhera and Watling's framework for integrating implicit bias recognition into health professions education[16] as our conceptual model, including these key recommendations: (1) create a safe space for learning, (2) emphasize how biased language impacts patient outcomes, (3) increase self-awareness of personal biases, (4) discuss strategies to overcome these patterns, and (5) enhance awareness of influence of implicit biases on others.

Our target audience was all health professionals caring for patients and documenting in the electronic medical record, including novice learners. There were no prerequisites for learners. We piloted the workshop with a diverse group of learners at three different professional conferences: the Stanford Medicine Second Annual Diversity and Inclusion Forum,[17] the Academy for Professionalism in Heath Care (APHC) Seventh Annual Meeting,[18] and the Academic Pediatric Association (APA) Region 9/10 Annual Meeting.[19] The LEAD program provided our workshop facilitators with trainings that included reducing unconscious bias and microaggressions and addressing privilege and allyship. We recommend that facilitators have experience with these topics. Audience participation was voluntary, and there were no incentives.

The workshop was designed to be 90 minutes, and course materials were inexpensive to duplicate. The facilitator's guide (Appendix A) provided a detailed agenda, and the PowerPoint presentation (Appendix B) contained detailed presenter notes below each slide. Key features of the workshop included the deliberate creation of a brave space,[20] a skit demonstrating biased language in a clinical presentation (Appendix C), a short didactic to define important terms, introduction of the Mindful Language Toolkit (Appendix D), group discussions, and opportunities to practice learned skills (Appendix E). Clinical cases can be adapted to the clinical discipline of the audience. However, we believe that the cases represent a wide variety of biased language commonly encountered in any discipline or position in health care.

All workshop attendees completed course evaluations (Appendix F) consisting of four 5-point Likert-style items (1 = *strongly disagree,* 5 = *strongly agree*), which were analyzed using descriptive statistics. Additionally, participants were asked four short-answer questions that were analyzed using the thematic content approach, which included data familiarization, identifying themes, developing and applying a coding scheme, and organizing codes and themes.[21] One investigator (Ria Pal) developed the coding framework, and two other investigators (Julia Raney and Kim Hoang) reviewed the coding scheme and made adjustments as necessary. This project was deemed exempt by the Stanford School of Medicine Institutional Review Board (Protocol #55660).

Copyright © 2021 Raney et al. This is an open-access publication distributed under the terms of the Creative Commons Attribution license.

Results

We collected course evaluations from 66 participants (12 APHC, 32 Stanford Diversity Day, and 22 APA conference attendees). Participants agreed that the workshop met its objectives ($M = 4.8$, $SD = 0.4$), was a valuable use of time ($M = 4.8$, $SD = 0.5$), and provided useful participant handouts and resources ($M = 4.6$, $SD = 0.7$). Most participants intended to apply information from the workshop in their future practice ($M = 4.8$, $SD = 0.4$; Table 1).

Table 1. Quantitative Evaluations ($N = 66$)

Statement[a]	*M*	*SD*	Range
The workshop met its objectives.	4.8	0.4	4-5
The workshop was a valuable use of my time.	4.8	0.5	3-5
The handouts included useful resources.	4.6	0.6	2-5
I will apply information learned today.	4.9	0.4	3-5

[a]Rated on a 5-point Likert scale (1 = *strongly disagree,* 5 = *strongly agree*).

Most participants found the sharing of personal stories, display of stigmatizing language on word clouds, and case-based examples to be particularly helpful activities (Table 2). Participants also enjoyed learning from group discussions with their colleagues and identified the Mindful Language Toolkit as a course highlight. Several participants offered feedback to better structure the workshop, including more time for small-group discussion. Many participants planned to teach lessons learned in the workshop to their colleagues and trainees, and some participants recommended adding dedicated workshop time to learn teaching and implementation strategies. Additionally, some participants suggested including more subtle and nuanced examples of bias and patient perspectives on the topic.

Participants planned to use the Mindful Language Toolkit, workshop handouts, or reflective exercises to practice language substitutions discussed in the presentation. However, participants also identified several barriers to change, including the rigid social structure in medicine, cultural norms, entrenched practice habits, normalization of stigmatizing language, and assumptions that colleagues or supervisors would lack interest in the topic. Time, fatigue, and burnout were also cited barriers. Interestingly, participants were concerned about staying caught up with appropriate terminology and being unaware of antibias language choices.

Discussion

Based on our experience piloting this course, we believe it fills an important gap by providing health professionals with a practical

Table 2. Themes From Open-Ended Course Evaluations ($N = 66$)

Theme	Subthemes	Illustrative Quotes
Changes to clinical practice	Incorporate mindful reflection and intentionality into own documentation	"Take a moment to consider the impact of your words when charting."<br>"[Ask myself] what would the patient interpret if they read my words."
	Share with colleagues, trainees, students, and faculty	"Incorporate teaching on biases and language into divisional or residency curriculum."<br>"Compassionately challenge colleagues on their language."
	Use Mindful Language Toolkit provided	"Replicate code of conduct card for colleagues."<br>"Use 'states' instead of claims."
Barriers to application of lessons learned	Entrenched habits and/or lack of interest from colleagues or superiors	"Difficult to change culture."<br>"Long held habits."
	Lack of social capital and skills needed to effect change	"I'm beginning third year rotations so my social capital is very low to influence care."<br>"How to provide constructive feedback to others without making them feel bad."
	Time, fatigue, burnout	"Burnout and how it leads to lack of empathy."<br>"Complacency."
	Staying caught up with appropriate unbiased terms	"Using the correct terms because language is always changing."
Most valuable aspects of workshop	Interactivity (sharing personal stories, word clouds, cased-based group work, large-group discussion)	"The examples shared by colleagues and the experiences they have faced."<br>"The discussion between providers of different backgrounds."<br>"Variety of teaching methods, including high tech approaches."
	Mindful Language Toolkit	"Pocket card and [toolkit] handout [helpful to prevent] forgetting."
	Facilitation of self-reflection	"Self-assessment of biases."<br>"Made me think critically about the language I use."
Improvements to workshop	Incorporate discussion of how to teach and address with colleagues	"As an educator, I would appreciate specific tips on how to teach this or address this when I see others falling into this pattern."<br>"How can we change the culture? People in attendance at this workshop are interested. How do you engage colleagues that may not be as aware and/or interested?"
	Provide more subtle bias and evidence-based examples	"More space for small groups to discuss nuances."
	Provide patient perspectives	"Hear from patients about their experience."
	More discussion time for small groups	"Perhaps more space for small groups to discuss nuances."

Copyright © 2021 Raney et al. This is an open-access publication distributed under the terms of the Creative Commons Attribution license.

framework to identify and replace biased language. Stigmatizing language is normalized in medicine, and providers require concrete skills to make meaningful changes to their long-standing language use. Most of our participants learned new actionable skills in the workshop that they intended to incorporate in their practices, such as avoiding labeling patients, reframing situations, and reflecting before documenting. Prior implicit bias research has shown that teaching intentional strategies, such as those in our workshop, is key to reducing biased behaviors following an intervention.[22] Our workshop offers antibias skills and strategies in the Mindful Language Toolkit and provides activities that allow participants to practice these techniques in a safe environment. However, the Mindful Language Toolkit should not stand alone without interactive teaching and facilitated discussion.

Participants described important barriers to the implementation of antibiased language in their workplaces. This feedback emphasizes the need to engage key stakeholders, such as unit supervisors and hospital administrators, who can facilitate normative practice changes. In response to this identified barrier, we lengthened the final group discussion to include strategies for building institutional support.

Participants offered several suggestions for how to overcome cultural barriers to practice change. For example, they recommended including discussion of how to educate colleagues about stigmatizing language. This topic is often included in implicit bias trainings[23,24] but was not initially covered in our workshop. However, given that social pressure is one of the most commonly cited barriers to addressing implicit bias, we modified our workshop to address this topic.

Lessons learned from piloting the workshop include the importance of providing ample time for practice of new skills and discussion with peer learners, as well as the broad application of the material to learners from different stages of training and discipline. The Mindful Language Toolkit is a critical feature of the training that summarizes key content and implementation strategies. We will extend the length of the workshop to better address potential barriers to change and to offer teaching techniques to participants interested in disseminating content to their colleagues and trainees.

Limitations

The setting of the workshop likely introduces some selection bias, as participants who attended may have been motivated to reduce disparities. Course evaluations focused on participants' self-reported reactions to the workshop, and we are unable to comment on knowledge or behavioral changes. One way to evaluate these in future studies would be to query electronic medical records for biased words such as *alcoholic* or *frequent flyer* following a training. In addition, course evaluations did not assess the impact of the interprofessional learning environment. Anecdotally, the presenters remember many interdisciplinary small groups and rich interprofessional discussions, so we believe the content translated well to our broad audience. Lastly, there was no presession assessment for pre/post comparisons, and we did not record the total number of participants, which would have allowed for a calculation of course evaluation response rate. We recommend that future course evaluations include presession surveys, the professions of the learners, and their experiences with the interprofessional education.

Conclusion

Learners in our workshop on stigmatizing language in health care were highly engaged and found the material clinically relevant. Participant feedback suggests that the workshop provided actionable skills for replacing biased language in clinical presentations and electronic health records. Barriers to practice change are important to address in future iterations of the workshop.

Appendices

A. Facilitator's Guide.docx

B. PowerPoint Presentation.pptx

C. Sign-out Skit.docx

D. Mindful Language Toolkit.docx

E. Clinical Cases.docx

F. Course Evaluation.docx

All appendices are peer reviewed as integral parts of the Original Publication.

Julia Raney, MD: Resident, Department of Pediatrics, Stanford University School of Medicine; ORCID: https://orcid.org/0000-0001-8579-9744

Ria Pal, MD: Resident, Division of Child Neurology, Department of Neurology and Neurological Sciences, Stanford University School of Medicine

Tiffany Lee, MD: Fellow, Department of Anesthesia, Stanford University School of Medicine

Samuel Ricardo Saenz, MD, MPH: Resident, Department of Psychiatry and Behavioral Sciences, Stanford University School of Medicine

Devika Bhushan, MD: Chief Medical Officer, California Office of the Surgeon General

Copyright © 2021 Raney et al. This is an open-access publication distributed under the terms of the Creative Commons Attribution license.

Peter Leahy, MD: Assistant Professor, Division of Genetics, Department of Pediatrics, Cook Children's Health Care System

Carrie Johnson, MBA: Stanford Pediatrics Residency Education Manager, Department of Pediatrics, Stanford University School of Medicine

Cynthia Kapphahn, MD, MPH: Clinical Professor, Division of Adolescent Medicine, Department of Pediatrics, Stanford University School of Medicine

Michael A. Gisondi, MD: Associate Professor, Department of Emergency Medicine, Stanford University School of Medicine

Kim Hoang, MD: Assistant Professor, Division of Pediatric Hospital Medicine, Department of Pediatrics, Stanford University School of Medicine

Acknowledgments
We wish to thank the Stanford University Leadership Education in Advancing Diversity (LEAD) program and Natalie Kirilcuk, MD, for their mentorship.

Disclosures
None to report.

Funding/Support
None to report.

Ethical Approval
The Stanford University School of Medicine Institutional Review Board approved this study.

References

1. Goddu AP, O'Conor KJ, Lanzkron S, et al. Do words matter? Stigmatizing language and the transmission of bias in the medical record. *J Gen Intern Med*. 2018;33(5):685-691. https://doi.org/10.1007/s11606-017-4289-2
2. Kelly JF, Westerhoff CM. Does it matter how we refer to individuals with substance-related conditions? A randomized study of two commonly used terms. *Int J Drug Policy*. 2010;21(3):202-207. https://doi.org/10.1016/j.drugpo.2009.10.010
3. Samulowitz A, Gremyr I, Eriksson E, Hensing G. "Brave men" and "emotional women": a theory-guided literature review on gender bias in health care and gendered norms towards patients with chronic pain. *Pain Res Manag*. 2018;2018:6358624. https://doi.org/10.1155/2018/6358624
4. Broyles LM, Binswanger IA, Jenkins JA, et al. Confronting inadvertent stigma and pejorative language in addiction scholarship: a recognition and response. *Subst Abuse*. 2014;35(3):217-221. https://doi.org/10.1080/08897077.2014.930372
5. Shattell MM. Stigmatizing language with unintended meanings: "persons with mental illness" or "mentally ill persons"? *Issues Ment Health Nurs*. 2009;30(3):199. https://doi.org/10.1080/01612840802694668
6. Dickinson JK, Guzman SJ, Maryniuk MD, et al. The use of language in diabetes care and education. *Diabetes Care*. 2017;40(12):1790-1799. https://doi.org/10.2337/dci17-0041
7. Hoffman KM, Trawalter S, Axt JR, Oliver MN. Racial bias in pain assessment and treatment recommendations, and false beliefs about biological differences between Blacks and Whites. *Proc Natl Acad Sci U S A*. 2016;113(16):4296-4301. https://doi.org/10.1073/pnas.1516047113
8. Schäfer G, Prkachin KM, Kaseweter KA, Williams AC. Health care providers' judgments in chronic pain: the influence of gender and trustworthiness. *Pain*. 2016;157(8):1618-1625. https://doi.org/10.1097/j.pain.0000000000000536
9. Goodyear K, Haass-Koffler CL, Chavanne D. Opioid use and stigma: the role of gender, language and precipitating events. *Drug Alcohol Depend*. 2018;185:339-346. https://doi.org/10.1016/j.drugalcdep.2017.12.037
10. Zestcott CA, Blair IV, Stone J. Examining the presence, consequences, and reduction of implicit bias in health care: a narrative review. *Group Process Intergroup Relat*. 2016;19(4):528-542. https://doi.org/10.1177/1368430216642029
11. Cooper A, Kanumilli N, Hill J, et al. Language matters. Addressing the use of language in the care of people with diabetes: position statement of the English Advisory Group. *Diabet Med*. 2018;35(12):1630-1634. https://doi.org/10.1111/dme.13705
12. Pont SJ, Puhl R, Cook SR, Slusser W; Section on Obesity; Obesity Society. Stigma experienced by children and adolescents with obesity. *Pediatrics*. 2017;140(6):e20173034. https://doi.org/10.1542/peds.2017-3034
13. Mayfield JJ, Ball EM, Tillery KA, et al. Beyond men, women, or both: a comprehensive, LGBTQ-inclusive, implicit-bias-aware, standardized-patient-based sexual history taking curriculum. *MedEdPORTAL*. 2017;13:10634. https://doi.org/10.15766/mep_2374-8265.10634
14. Stagno S, Crapanzano K, Schwartz A. Keeping the patient at the center: teaching about elements of patient-centered care. *MedEdPORTAL*. 2016;12:10500. https://doi.org/10.15766/mep_2374-8265.10500
15. Leadership Education in Advancing Diversity. Stanford Medicine. Accessed April 1, 2020. https://med.stanford.edu/pediatrics/education/events/lead.html
16. Sukhera J, Watling C. A framework for integrating implicit bias recognition into health professions education. *Acad Med*. 2018;93(1):35-40. https://doi.org/10.1097/ACM.0000000000001819
17. Bhushan D, Leahy P, Lee T, et al. Words hurt: identifying and removing stigmatizing language from clinical settings. Workshop presented at: Stanford Medicine Annual Diversity and Inclusion Forum; May 10, 2019; Stanford, CA.
18. Lee T, Bhushan D, Leahy P, et al. Words hurt: identifying and removing stigmatizing language from clinical settings. Workshop presented at: Academy for Professionalism in Health Care Annual Meeting; May 15-17, 2019; New Orleans, LA.

Copyright © 2021 Raney et al. This is an open-access publication distributed under the terms of the Creative Commons Attribution license.

19. Raney J, Saenz SR, Kapphahn C, et al. “The sickler in room 202 is a poor historian”: exploring how stigmatizing language reflects implicit bias. Workshop presented at: Academic Pediatric Association Region 9/10 Annual Meeting; Spring 2019; Monterey, CA.

20. Ali D. *Safe Spaces and Brave Spaces: Historical Context and Recommendations for Student Affairs Professionals*. National Association of Student Personnel Administrators; 2017. NASPA Policy and Practice Series no. 2.

21. Pope C, Ziebland S, Mays N. Analysing qualitative data. *BMJ*. 2000;320(7227):114-116. https://doi.org/10.1136/bmj.320.7227.114

22. FitzGerald C, Martin A, Berner D, Hurst S. Interventions designed to reduce implicit prejudices and implicit stereotypes in real world contexts: a systematic review. *BMC Psychol*. 2019;7:29. https://doi.org/10.1186/s40359-019-0299-7

23. Maurer MJ, Goldstein RL, Riveros LFV, et al. Death by a thousand paper cuts: microaggressions and their effects on the learning climate. Workshop presented at: Association of Pediatric Program Directors Annual Spring Meeting; March 20-23, 2018; Atlanta, GA.

24. Acholonu RG, Cook TE, Roswell RO, Greene RE. Interrupting microaggressions in health care settings: a guide for teaching medical students. *MedEdPORTAL*. 2020;16:10969. https://doi.org/10.15766/mep_2374-8265.10969

Received: July 27, 2020
Accepted: December 20, 2020
Published: March 2, 2021

Copyright © 2021 Raney et al. This is an open-access publication distributed under the terms of the Creative Commons Attribution license.
